# Global associations between regional gray matter volume and diverse complex cognitive functions: evidence from a large sample study

**DOI:** 10.1038/s41598-017-10104-8

**Published:** 2017-08-30

**Authors:** Hikaru Takeuchi, Yasuyuki Taki, Rui Nouchi, Ryoichi Yokoyama, Yuka Kotozaki, Seishu Nakagawa, Atsushi Sekiguchi, Kunio Iizuka, Yuki Yamamoto, Sugiko Hanawa, Tsuyoshi Araki, Carlos Makoto Miyauchi, Takamitsu Shinada, Kohei Sakaki, Yuko Sassa, Takayuki Nozawa, Shigeyuki Ikeda, Susumu Yokota, Magistro Daniele, Ryuta Kawashima

**Affiliations:** 1 Division of Developmental Cognitive Neuroscience, Institute of Development, Aging and Cancer, Tohoku University, Sendai, Japan; 2 Division of Medical Neuroimaging Analysis, Department of Community Medical Supports, Tohoku Medical Megabank Organization, Tohoku University, Sendai, Japan; 3 Department of Radiology and Nuclear Medicine, Institute of Development, Aging and Cancer, Tohoku University, Sendai, Japan; 4 Creative Interdisciplinary Research Division, Frontier Research Institute for Interdisciplinary Science, Tohoku University, Sendai, Japan; 5 Human and Social Response Research Division, International Research Institute of Disaster Science, Tohoku University, Sendai, Japan; 6 Smart Ageing International Research Center, Institute of Development, Aging and Cancer, Tohoku University, Sendai, Japan; 7 School of Medicine, Kobe University, Kobe, Japan; 8 Division of Clinical research, Medical-Industry Translational Research Center, Fukushima Medical University School of Medicine, Fukushima, Japan; 9 Department of Functional Brain Imaging, Institute of Development, Aging and Cancer, Tohoku University, Sendai, Japan; 10 Department of Psychiatry, Tohoku Pharmaceutical University, Sendai, Japan; 11 Department of Psychosomatic Research, National Institute of Mental Health, National Center of Neurology and Psychiatry, Kodaira, Tokyo, Japan; 12 Department of Psychiatry, Tohoku University Graduate School of Medicine, Sendai, Japan; 13 Graduate School of Arts and Sciences, Department of General Systems Studies, The University of Tokyo, Tokyo, Japan; 14 Department of Ubiquitous Sensing, Institute of Development, Aging and Cancer, Tohoku University, Sendai, Japan; 15 School of Electronic, Electrical and Systems Engineering, Loughborough University, Loughborough, England

## Abstract

Correlations between regional gray matter volume (rGMV) and psychometric test scores have been measured to investigate the neural bases for individual differences in complex cognitive abilities (CCAs). However, such studies have yielded different rGMV correlates of the same CCA. Based on the available evidence, we hypothesized that diverse CCAs are all positively but only weakly associated with rGMV in widespread brain areas. To test this hypothesis, we used the data from a large sample of healthy young adults [776 males and 560 females; mean age: 20.8 years, standard deviation (SD) = 0.8] and investigated associations between rGMV and scores on multiple CCA tasks (including non-verbal reasoning, verbal working memory, Stroop interference, and complex processing speed tasks involving spatial cognition and reasoning). Better performance scores on all tasks except non-verbal reasoning were associated with greater rGMV across widespread brain areas. The effect sizes of individual associations were generally low, consistent with our previous studies. The lack of strong correlations between rGMV and specific CCAs, combined with stringent corrections for multiple comparisons, may lead to different and diverse findings in the field.

## Introduction

The complexity of cognitive tasks has been shown to affect a number of important variables in psychology. Performance on more complex cognitive tasks is more strongly associated with intelligence, circadian rhythm, and age-related variance (for summary, see ref. 1). The complexity of tasks in this context is traditionally associated with the number of processing operations required to perform the task and the consequent burden on working memory^2^. Complex cognitive abilities (CCAs) include non-verbal reasoning^3^, which is well correlated with general intelligence (g factor), which in turn is predictive of performance on a wide range of cognitive tasks^4^, capacity of working memory which is the limited capacity storage system involved in the maintenance and manipulation of information over short periods of time^5^, and executive functions, which refer to cognitive processes involved in the intentional component of environmental interaction, including planning and inhibitory control^6^. Another common metric of cognitive ability is simple processing speed, which refers to the speed with which individuals execute elementary cognitive tasks. However, it is widely believed that the speed with which individuals can execute more complex cognitive tasks (refer to as complex processing speed in this study) show distinct psychological characteristics. For example, complex processing speed (PS) is more strongly associated with psychometric intelligence^7^, aging^2^, and inhibitory cognitive processes^8^.

One method that has been used to reveal the neural basis of these individual differences in CCAs is brain morphometry, specifically the associations between cortical structure (e.g., volume or thickness) and CCA scores. Indeed, numerous studies have investigated gray matter structural correlates of psychometric intelligence (for review, see ref. 9, for a meta-analysis, see ref. 10), although relatively few have investigated gray matter structural correlates of certain CCAs such as WMC^11^ and attention/inhibition component of executive function^12^. However, findings have been highly inconsistent. In a review of these results^9^, it was concluded that although some areas in the frontal lobe (Brodmann area 10) and parietal lobe (Brodmann area 39, 40) were often significantly associated with psychometric intelligence (50% of studies), almost all brain areas analyzed showed some level of correlation with psychometric intelligence in at least some studies^13^. Although a meta-analysis^10^ found significant correlations between psychometric intelligence and gray matter structure in several areas, these findings did not cover the entire brain and may be partly incongruent with previous studies. This lack of consistency is also seen in studies focusing on WMC and regional brain structure. Correlations were identified between cortical structures and WMC in the anterior cingulate and frontopolar area^14^, distributed areas in the neocortex and subcortical areas^15^, and dorsolateral prefrontal cortex^11^, although different methods were applied in these studies. Therefore, there is no comprehensive model of rGMV–CCA correlations that can explain these previous inconsistent findings.

A previous study suggested that these discrepancies are partly attributed to different preprocessing methods^13^. This study found that different preprocessing methods led to divergent results in a sample of approximately 40 subjects; however, even if the same method was applied, results differed in the replication sample. Therefore, the sources of these inconsistencies are currently unclear. However, many previous studies also included relatively small sample sizes (N < 100) compared to more recent structural studies and thus lacked statistical power. In addition to low effect size and statistical power, it is also possible that the multiple areas showing significant associations with CCA in different studies are in fact functionally associated with diverse CCAs. A series of recent studies using huge sample sizes (N > several hundred) of young adults showed that associations between individual aspects of cognition and regional gray matter structure were generally low^16^. Moreover, our previous study on simple processing speed involving several hundred subjects showed that performance was positively correlated with widespread regional white matter volume (rWMV) across different simple processing speed tasks; furthermore, the strengths of the associations were generally low and no single area showed significant specific correlations between rWMV and simple processing speed^17^. Similarly, a previous meta-analysis utilizing the data of more than 1500 subjects showed only a small correlation between total brain volume and psychometric intelligence (r = 0.33)^18^. Considering that rGMV tends to be well explained by global signal variation and performance on different cognitive tasks by shared variance^19^, the rGMV of widespread areas may correlate better with CCAs than more restricted regions. Based on these considerations, we hypothesized that diverse CCAs are all positively associated with rGMV in widespread areas but that each association is weak.

The purpose of this study was to test our hypothesis by investigating the relationships between rGMV and multiple CCAs in a huge sample. Therefore, we analyzed rGMV data from a large sample of young adults (n > 1000). Considering the effects of preprocessing methods on results of structural studies^13^, we employed the newest version of voxel-based morphometry (VBM)^20^. Further, the difficulty of properly correcting the multiple comparisons in whole brain rGMV analyses have been suggested^21^ and permutation-based methods have been shown to quite accurately correct for multiple comparisons in rGMV studies^21, 22^. And, the present study also utilized rigorous permutation-based statistical methods using threshold-free cluster enhancement^23^.

The anatomical correlates of human CCAs are of great interest, and numerous studies have attempted to identify specific brain regions underlying CCA^9^. To date, however, findings have been inconsistent. The principal aim of the present study was to provide a comprehensive explanation for these unresolved inconsistencies with robust statistical power using rigorous statistical methods.

## Methods

### Subjects

In total, 1336 healthy, right-handed individuals (776 men and 560 women) participated in this study as part of our ongoing project to investigate the associations among brain imaging, cognitive functions and aging^24–26^. Descriptions in this subsection were mostly reproduced from our previous study^27^. Data from a portion of subjects were used in our previous studies to investigate the associations between Stroop interference and gray matter structures^12^. Some of the subjects who took part in this study also became subjects of our intervention studies (psychological data and imaging data recorded before the intervention were used in this study)^28^. In this project’s experiment, psychological tests and MRI scans not described in this study were performed together with those described in this study. The mean age of subjects was 20.8 years (standard deviation [SD], 1.8). All subjects were university, college, or postgraduate students or subjects who had graduated from these institutions within 1 year before the experiment and had normal vision. None had a history of neurological or psychiatric illness. Handedness was evaluated using the Edinburgh Handedness Inventory^29^. Written informed consent was obtained from each subject in accordance with the Declaration of Helsinki (1991). This study was approved by the Ethics Committee of Tohoku University. All experiments were performed in accordance with the institutional guidelines and regulations.

### Psychological measures

For evaluation of the cognitive functions, a battery of neuropsychological tests was administered. This battery included the following contents: [A] Raven’s Advanced Progressive Matrices^3^, a nonverbal reasoning task to measure non-verbal reasoning ability. [B] A (computerized) digit span task, a verbal WM task to measure WMC (for the detail of this task, see ref. 30). [C] The Stroop task (Hakoda’s version)^31^, which measures the attention/inhibition component of executive functions. The test involved a control task (Color-Word task) and a Stroop task. Stroop interference rates are calculated as follows:

Stroop interference = (correct answers of Color-word test − correct answers of Stroop test)/(correct answers of Color-word test) × 100. This Stroop interference rate is the standard measure for this test and its reliability has been established^31^. [**D**] Tanaka B-type intelligence test^32^. Type 3B, which was a nonverbal mass intelligence test, was used in this study. The test consists of three sub factors: perceptual, spatial, and reasoning. The tasks for these latter two factors require relatively more complex cognitive judgments so the scores were treated as indices of complex PS. The data of subjects who misunderstood the rules of the parts of the tests were excluded from the tasks.

### Image acquisition

The methods for MR image acquisition were described in our previous study^27^. All MRI data acquisition was performed using a 3-T Philips Achieva scanner. High-resolution T1-weighted structural images (T1WIs: 240 × 240 matrix, TR = 6.5 ms, TE = 3 ms, FOV = 24 cm, slices = 162, slice thickness = 1.0 mm) were collected using a magnetization-prepared rapid gradient echo sequence.

### Pre-processing of structural data

Preprocessing of the structural data was performed using Statistical Parametric Mapping software (SPM12; Wellcome Department of Cognitive Neurology, London, UK) implemented in Matlab (Mathworks Inc., Natick, MA, USA). Using the new segmentation algorithm implemented in SPM12, T1-weighted structural images of each individual were segmented into 6 tissues. In this new segmentation process, default parameters were used, except that the Thorough Clean option was used to eliminate any odd voxel, affine regularization was performed with the International Consortium for Brain Mapping template for East Asian brains, and the sampling distance was set at 1 mm. We then proceeded to the diffeomorphic anatomical registration through exponentiated lie algebra (DARTEL) registration process implemented in SPM12. We used DARTEL import images of the 2 TPMs from the abovementioned new segmentation process. First, the template for the DARTEL procedures was created using imaging data from 800 participants (400 males and 400 females). The following methods were the same as in our previous study and descriptions were reproduced from our previous study^33^. Next, using this existing template, the DARTEL procedures were performed for all of the subjects in the present study. In these procedures, default parameter settings were used. The resulting images were spatially normalized to the Montreal Neurological Institute (MNI) space to give images with 1.5 × 1.5 × 1.5 mm^3^ voxels. In addition, we performed a volume change correction (modulation) by modulating each voxel with the Jacobian determinants derived from spatial normalization, which allowed us to determine regional differences in the absolute amount of brain tissue^20^. Subsequently, all images were smoothed by convolving them with an isotropic Gaussian kernel of 8 mm full width at half maximum (FWHM).

### Group-level statistical analysis

Statistical analyses relating to rGMV were performed using SPM8 software. SPM8 was used for the compatibility of the software that we used for permutation-based statistics which is described below, as well as the script we used for the statistical analyses. We included only voxels with an rGMV signal intensity of >0.05. The GM value threshold of 0.05 is a widely used value that has been reported in numerous previous VBM studies^34–41^.

In the whole-brain multiple regression analyses, we tested for relationships between each cognitive function and rGMV. In all of the analyses, the dependent variables were the rGMV signal values at each voxel. There were two types of analyses for each cognitive function, and as a result, 12 individual analyses. One type did not correct for global signals (total gray matter volume) while the other did. The former analyses include age, sex, and one of the following: (A) RAPM score, (B) digit span score, (C) Stroop interference (in the case of this variable only, performance on a control Color-Word task was included as an additional covariate as in a previous study)^12^, (D) spatial factor of the Tanaka B-type intelligence test (TBIT), (E) reasoning factor of the TBIT, or (F) perceptual factor of the TBIT. The latter analyses included total gray matter volume as well as age, sex, and one of (A)−(F).

The rationale for not performing analyses that do not correct for global signals was previously described in detail^40^. Basically, when the GMV of extensive brain regions correlates with target variables, it is inappropriate to regress out the global signal. This is because we were interested in the absolute difference (compared with the relative difference between imaging measures such as rGMV of one area and those of other brain regions) with regard to the complex cognitive abilities (Mechelli *et al*., 2005, Current Medical Imaging Reviews). Removing the global differences would correspond to assuming that the total GMV (etc.) would be the same in all participants, and in this case, rGMV represents the relative difference compared with other areas. We did not include environmental factors (or genetic factors) that could form the associations between cognitive functions and cortical structures (such as social economic status) like most of previous studies of the associations between cognitive functions and brain structures. This is because these factors are not confounding variables, and our interest lies in the associations between cognitive functions and cortical structures regardless of how environmental and genetic factors form them. We did not perform a single multiple regression analysis that included scores of all cognitive tests at once. This is because for example, working memory performance and general intelligence are believed to share some fundamental cognitive processes (Colom, *et al*., 2007; Conway, Kane, and Engle, 2003) and we believe regressing out the effect of general intelligence when we look at the associations between working memory performance and rGMV does not make sense. The same things hold true to the other combinations such as relationship between the reasoning task’s performance and performance of complex processing speed involving reasoning. Sex differences of anatomical correlates of CCA have nothing to do the purpose of this study as can be seen in Introduction.

A multiple comparison correction of the cross-sectional analyses was performed using threshold-free cluster enhancement (TFCE)^23^, with randomized (5,000 permutations) nonparametric permutation testing via the TFCE toolbox (http://dbm.neuro.uni-jena.de/tfce/). We applied the threshold of an FWE corrected *P* < 0.05. According to Smith and Nichols^23^, the method of TFCE takes a raw statistical image and produces an output image in which the voxel-wise values (TFCE values) represent the amount of cluster-like local spatial support. In this method, the new value of each voxel is given by the sum of the “scores” of all “supporting sections” underneath it, and the score of each section is simply its height multiplied by its extent. The output value is therefore a weighted sum of all local clustered signals, without the need for hard cluster-forming thresholding. For inference, the TFCE image can easily be converted into voxel-wise p-values (either uncorrected or corrected for multiple comparisons across space) via permutation testing.

### Effects of g factor on rGMV and of “g-independent” score for each test on rGMV

Although we primarily focused on individual cognitive measures in this study, several previous studies have assessed the correlation of gray matter structures with the general intelligence factor (g factor, a factor that partially explains success in diverse forms of cognitive activity) as well as specific effects that are not explained by g factor^42^.

In order to estimate the effects of g factor as well as task-specific effects independent of g factor on rGMV for each task, we first performed exploratory factor analysis.

Data were analyzed using SPSS 22.0 statistical software (SPSS Inc., Chicago, IL). Promax-rotated factor analysis (unweighted least squares method) of scores for each question in six cognitive tasks was performed. Two factors that showed eigen values higher than 1 were extracted based on the Kaiser–Guttman criterion^43^.

The factor loadings of each task to the first factor were as follows: RAPM: 0.579, digit span: 0.465, Stroop interference: −0.006, spatial factor of TBIT: 0.685, reasoning factor of TBIT: 0.593, and perceptual factor of TBIT: 0.634.

The factor loadings of each task to the second factor were as follows: RAPM: −0.033, digit span: −0.038, Stroop interference: 0.999, spatial factor of TBIT: 0.018, reasoning factor of TBIT: 0.045, and perceptual factor of TBIT: −0.004.

The correlation coefficient between the first and second factors was 0.034.

As can be seen, Stroop interference showed a rather distinct pattern. This may be due to the nature of Stroop interference in which “more and faster” does not necessarily lead to better performance. Thus, in subsequent g-related analyses. Stroop interference was excluded from the analyses.

To calculate the g-independent score for each test, we followed the method of Karama *et al*.^42^. We computed five different g scores in order to estimate “g-independent” scores for each test. Each of these scores was based on only four tests instead of the five available to avoid inadvertently controlling for the contribution of the test of interest when controlling for g. For instance, “RAPM”-free g scores were computed from four tasks excluding the “RAPM” test. One regression analysis was conducted for each test, using the appropriate g score as the independent variable and the respective specific test score as the dependent variable. Residuals from these regression analyses were considered to represent “g-independent” specific test scores.

In whole-brain multiple regression analyses, we tested for relationships between calculated cognitive measures and rGMV. In all analyses, the dependent variables were the rGMV signal values at each voxel. Six whole-brain multiple regression analyses were performed, all of which included age and sex as covariates. Additionally, six analyses included the following dependent variables: (1) g factor calculated from the five tasks described above, (2) g-independent RAPM score and g factor calculated from the other four tasks, (3) g-independent digit span score and g factor calculated from the other four tasks, (4) g-independent spatial factor of TBIT and g factor calculated from the other four tasks, (5) g-independent reasoning factor of TBIT and g factor calculated from the other four tasks, and (6) g-independent perceptual factor of TBIT and g factor calculated from the other four tasks. The remaining statistical analyses were performed as described for the main analyses of each score.

## Results

### Basic data

The average, SD, and range of RAPM scores, digit span scores, Stroop interference scores, TBIT subscores for spatial, reasoning, and perceptual factors, and the ages of males and females in our sample are presented in Table 1. The associations between performance on specific CCA tasks and total GMV were tested by partial regression analyses. Total GMV was significantly correlated with the digit span score [standardized beta coefficient (β) = 0.101, P < 0.001], Stroop interference (β = −0.080, P < 0.001), and TBIT spatial (β = 0.088, P < 0.001) and reasoning factor (β = 0.074, P < 0.01) scores, but not with the RAPM score (β = 0.032, P > 0.05) and TBIT perceptual factor score (β = 0.032, P > 0.05). All task performances were significantly correlated with one another. For statistical values, see Table 2.

**Table 1:** Average, SD, and range of cognitive test scores and ages of males and females in our sample.

	Male			Female		
	average	SD	range	average	SD	range
Age in years (M:776, F:560)	20.87	1.88	18–27	20.70	1.62	18–27
RAPM score (M:776, F:560)	28.7	3.83	13–36	28.03	3.83	15–36
digit span (M:733, F:544)	37.05	7.19	13–67	35.03	6.44	17–58
Stroop interference (M:771, F:559)	0.072	0.090	−0.24–0.46	0.063	0.086	−0.25–0.386
spatial factor of TBIT (M:668, F:495)	43.94	5	28–57	41.66	4.76	28–55
reasoning factor of TBIT (M:668, F:495)	20.94	2.74	11–30	19.25	2.78	10–28
perceptual factor of TBIT (M:666, F:491)	49.38	7.31	21–72	49.36	6.62	33–71

RAPM, Raven’s Advanced Progressive Matrix; TBIT, Tanaka B-type intelligence test.

**Table 2:** Partial regression coefficients among cognitive tasks corrected for age and sex (in the case of Stroop interference, regression was corrected for control task performance in addition to age and sex).

	1	2	3	4	5	6	7
1. RAPM score	—						
2. digit span	0.270 (1.028 × 10^−22^)	—					
3. Stroop interference	−0.098 (3.421 × 10^−4^)	−0.140 (6.076 × 10^–7^)	—				
4. spatial factor of TBIT	0.386 (1.831 × 10^−42^)	0.273 (3.243 × 10^−21^)	−0.098 (0.001)	—			
5. reasoning factor of TBIT	0.347 (2.997 × 10^−34^)	0.266 (3.425 × 10^−20^)	−0.061 (0.038)	0.368 (1.272 × 10^−38^)	—	—	
6. perceptual factor of TBIT	0.356 (8.324 × 10^−36^)	0.289 (1.667 × 10^−23^)	−0.202 (4.041 × 10^−12^)	0.481 (5.131 × 10^−68^)	0.379 (9.549 × 10^−41^)	—	
7. total gray matter volume	0.032 (0.166)	0.101 (1.669 × 10^−5^)	−0.080 (0.001)	0.088 (4.022 × 10^−4^)	0.074 (0.004)	0.032 (0.194)	—

Before whole-brain analyses, it should be noted that total GMV strongly globally correlated with rGMV. A whole-brain multiple regression analysis of the entire sample corrected for the effects of age and sex revealed that total GMV was significantly correlated with rGMV in 532433 out of 532464 voxels in the whole brain [P < 0.05, corrected for F.W.E (TFCE)]. The beta estimates of the associations between the mean rGMV and each cortical ROI were all at least 0.5 in the multiple regression analyses correcting for the effects of age and sex, while the beta estimates of the associations between the mean TMV and each subcortical ROI were all at least 0.3 in the multiple regression analyses correcting for the effects of age and sex.

### Relationships between non-verbal reasoning performance and rGMV

After correcting for effects of age and sex, a whole-brain multiple regression analysis revealed no significant correlations between RAPM score and rGMV. However, trends were found in brain areas showing significant correlations in a previous study that used similar methods (Raven’s standard progressive matrix test, controlling for effects of age and sex) and a large sample (N = 296) of young Asian adults^44^. In the present study, positive trends for RAPM and rGMV were found in the left occipital lobe (x, y, z = −27, −103.5, −10.5, P = 0.057, corrected for multiple comparisons (FWE)), right anterior temporal lobe (x, y, z = 39, −1.5, −28.5, P = 0.059, corrected for multiple comparisons (FWE)), and right frontoinsular cortex (x, y, z = 46.5, 3, 3, P = 0.084, corrected for multiple comparisons (FWE)).

After correcting for the effects of age, sex, and total GMV, whole-brain multiple regression analyses revealed no significant correlations between RAPM score and rGMV.

### Relationships between verbal WMC and rGMV

**Figure 1:**
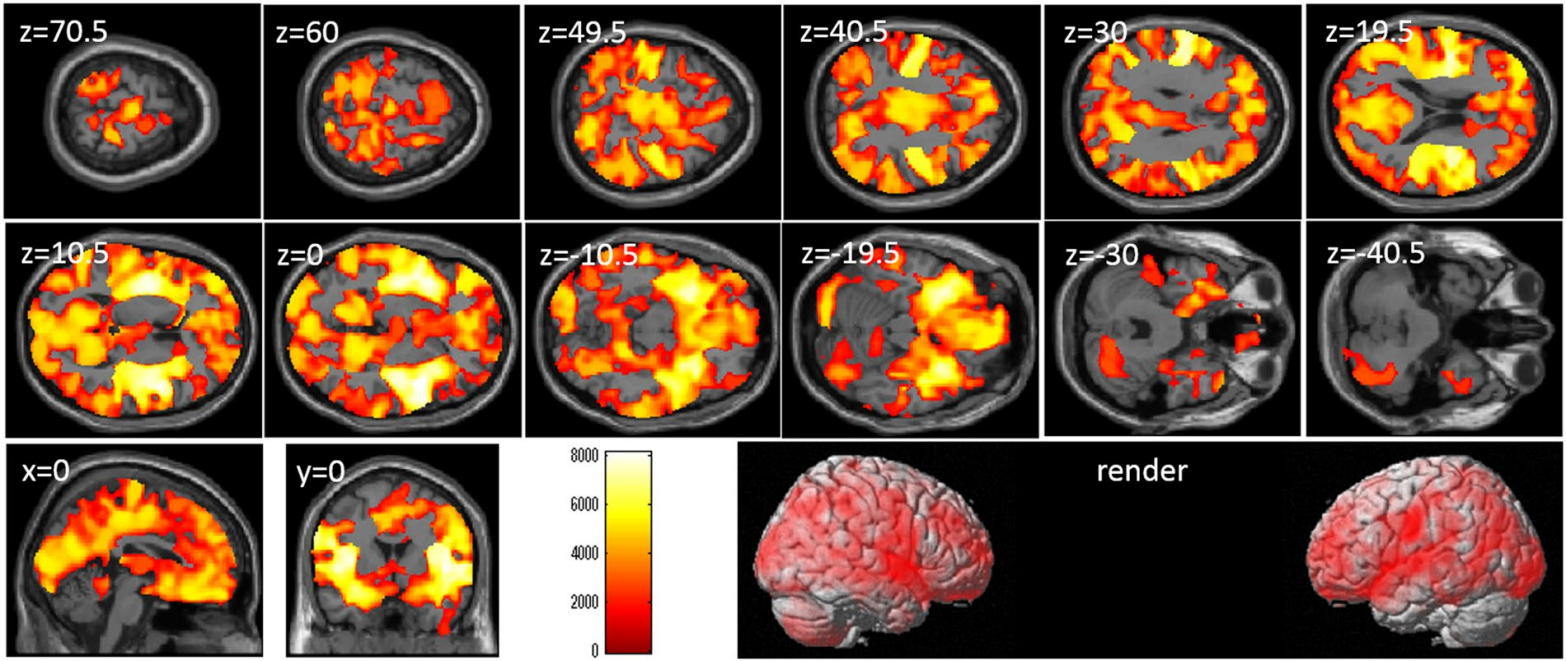
Relationships between verbal working memory (WM) and regional gray matter volume (rGMV). Individual panels represent areas of significant positive correlation between the digit span score and rGMV by multiple regression analysis corrected for effects of age and sex. The results shown were obtained using a threshold of TFCE, *P* < 0.05 corrected (FWE) based on 5000 permutations. The color bar represents the threshold-free cluster enhancement (TFCE) values. Significant positive correlations were found across the whole brain.

After correcting for effects of age and sex, whole-brain multiple regression analyses revealed significant correlations between digit span score and rGMV across a wide range of brain areas (Table 3 and Fig. 1). The labeling of anatomical gray matter regions based on the automated anatomical labeling (AAL) atlas option of the WFU PickAtlas Tool revealed voxels of significant correlation in all brain regions.

**Table 3:** Brain regions exhibiting significant correlations between digit span scores and rGMV.

No	Included gray matter areas* (number of significant voxels in left and right side of each anatomical area)	x	y	z	TFCE value	Corrected *p*-value (TFCE, FWE)	Cluster size (voxel)
1	Amygdala (L:517, R:562)/Angular gyrus (L:2381, R:3178)/Calcarine Cortex (L:4958, R:3746)/Caudate (L:814, R:771)/Anterior cingulum (L:3034, R:2120)/Middle cingulum (L:4103, R:4061)/Posterior cingulum (L:969, R:725)/Cuneus (L:2719, R:2779)/Inferior frontal operculum (L:2146, R:2404)/Inferior frontal orbital area (L:3456, R:2550)/Inferior frontal triangular (L:3797, R:2277)/Middle frontal medial area (L:1681, R:2115)/Middle frontal orbital area (L:1606, R:682)/Middle frontal other areas (L:6548, R:7375)/Superior frontal medial area (L:5859, R:3199)/Superior frontal orbital area (L:1698, R:1112)/Superior frontal other areas (L:4824, R:4604)/Fusiform gyrus (L:1458, R:3077)/Heschl gyrus (L:560, R:587)/Hippocampus (L:1382, R:1774)/Insula (L:4550, R:4424)/Lingual gyrus (L:3340, R:3561)/Inferior occipital lobe (L:1516, R:1264)/Middle occipital lobe (L:5276, R:3871)/Superior occipital lobe (L:1806, R:2121)/Pallidum (L:248, R:186)/Paracentral lobule (L:1314, R:1446)/Parahippocampal gyrus (L:1097, R:1794)/Inferior parietal lobule (L:3707, R:2758)/Superior parietal lobule (L:3238, R:2361)/Postcentral gyrus (L:6951, R:5398)/Precentral gyrus (L:3594, R:5602)/Precuneus (L:5756, R:6224)/Putamen (L:1350, R:1711)/Rectus gyrus (L:2073, R:1843)/Rolandic operculum (L:2310, R:3116)/Supplemental motor area (L:2801, R:3964)/Supramarginal gyrus (L:1996, R:3795)/Inferior temporal gyrus (L:2837, R:2555)/Middle temporal gyrus (L:7377, R:6997)/Temporal pole (L:2940, R:2929)/Superior temporal gyrus (L:4952, R:5751)/Thalamus (L:555, R:1432)/Cerebellum (L:1915, R:8815)/	45	6	−1.5	8144.59	0.0002	305663
2	None	−12	−1.5	30	1507.24	0.042	4

*Labeling of anatomical gray matter regions was based on the WFU PickAtlas Tool (http://www.fmri.wfubmc.edu/cms/software#PickAtlas/)^68, 69^ and the PickAtlas automated anatomical labeling atlas option^70^. Temporal pole and cerebellum include all subregions of these areas in the atlas.

The observed effect sizes of the associations between CCA and rGMV for each anatomical area are presented in Supplemental Table 1. The maximum beta values were around 0.15 for the associations between rGMV in the insula and digit span score. We have also provided r values of simple regression analyses for each male and female sample individually (Supplemental Table 2).

**Figure 2:**
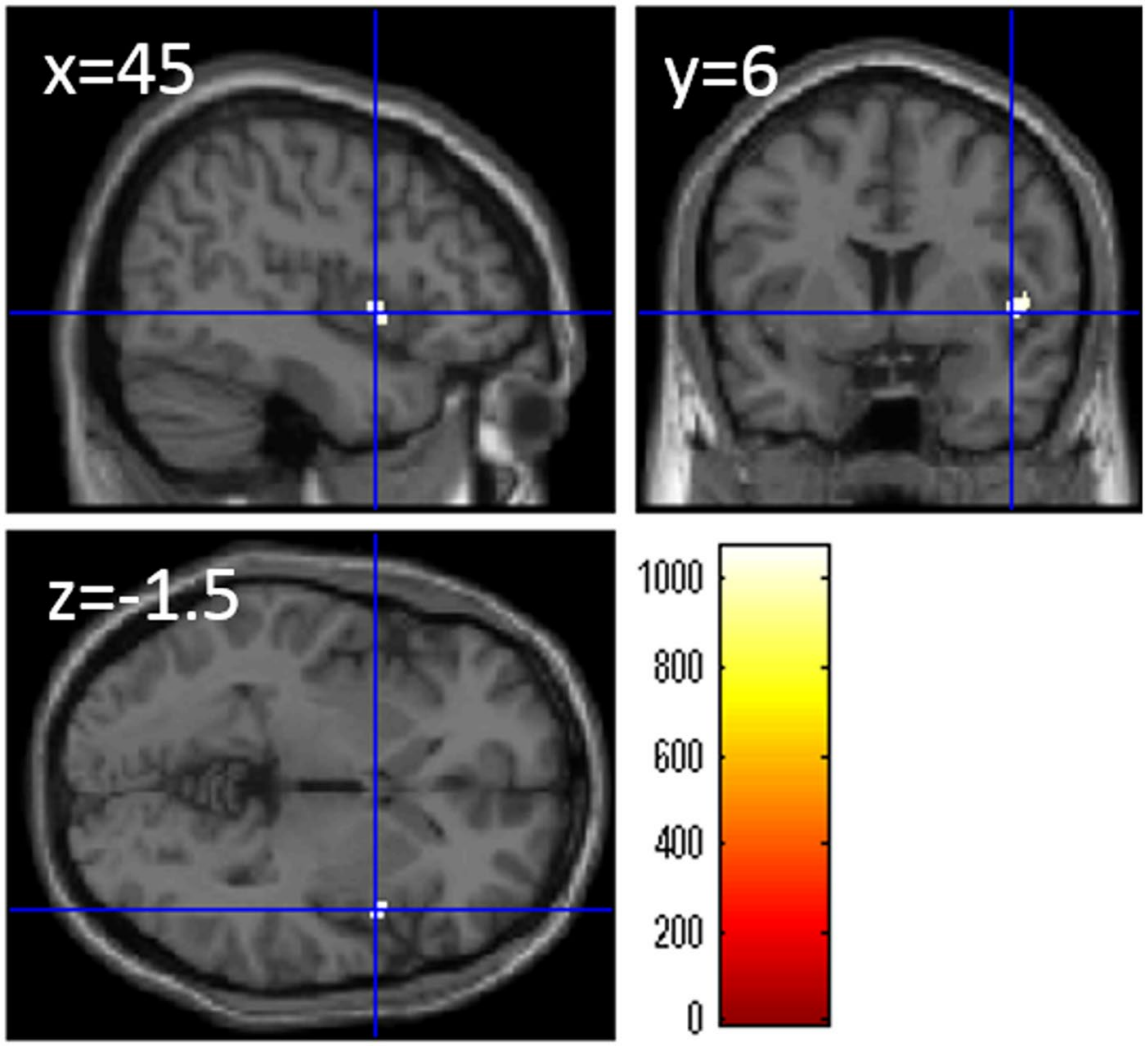
Relationships between verbal WM capacity (WMC) and rGMV after regressing out the effects of total gray matter volume. Panels show areas of significant positive correlation between the digit span score and rGMV by multiple regression analysis corrected for effects of age, sex, and total gray matter volume. The results shown were obtained using a threshold of TFCE, *P* < 0.05 corrected (FWE) based on 5000 permutations. The color bar represents the TFCE values in section images. Significant positive correlations were found in the right insula.

After correcting for effects of age, sex, and total GMV, whole-brain multiple regression analyses revealed a significant correlation in the right insula (x, y, z = 45, 6, −1.5, TFCE value = 1074.23, P = 0.035, corrected for multiple comparisons (FWE), 68 voxels in this area, Fig. 2).

### Relationships between rGMV and performance on the attention/inhibition component of executive function

**Figure 3:**
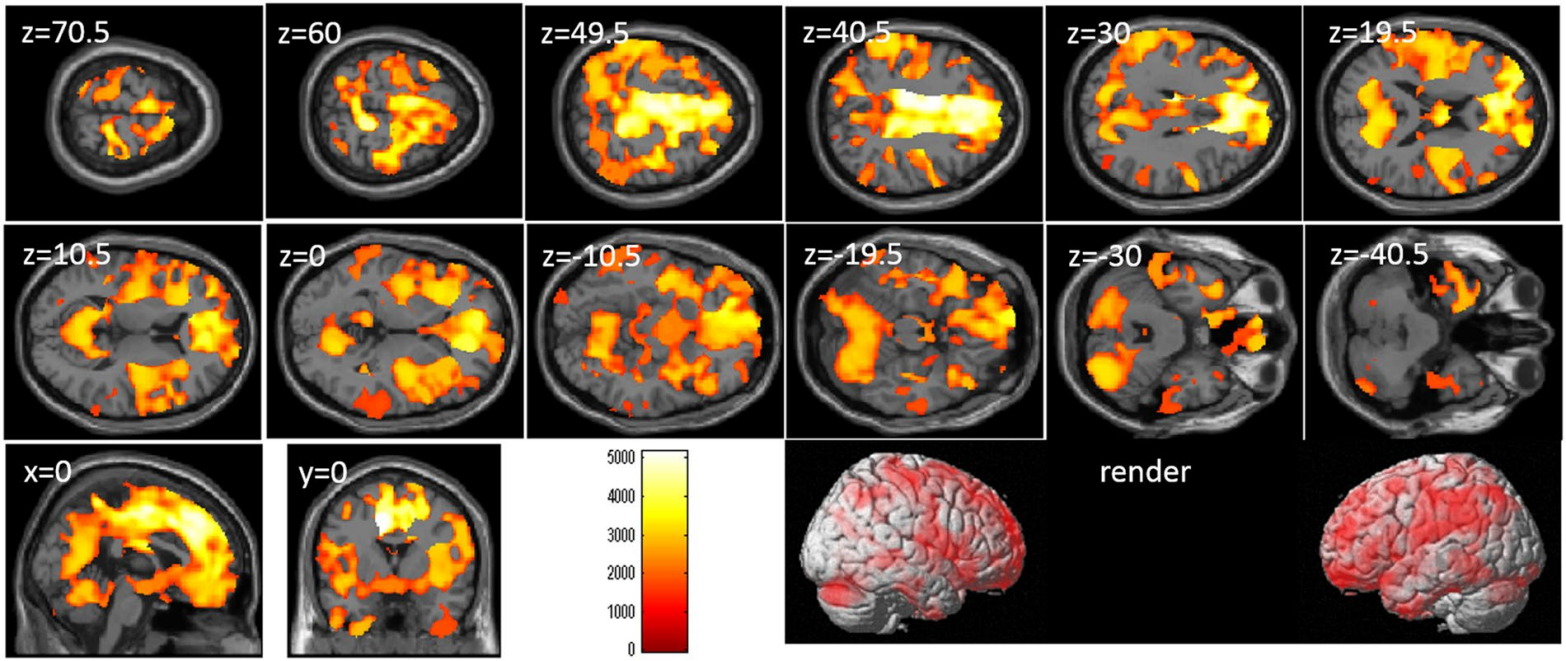
Relationships between rGMV and performance on tasks of the attention/inhibition component of executive function. Panels show areas of significant negative correlation between Stroop interference and rGMV by multiple regression analysis corrected for effects of age, sex, and control task performance (Color-Word task). The results shown were obtained using a threshold of TFCE, *P* < 0.05 corrected (FWE) based on 5000 permutations. In section images, the color bar represents the TFCE value. Significant positive correlations were found across the whole brain.

After correcting for the effects of age, sex, and control task performance (Color-Word task), whole-brain multiple regression analyses revealed significant negative correlations between Stroop interference score and rGMV across a wide range of brain areas (Table 4 and Fig. 3). In all brain regions of the AAL atlas, there were voxels of significant correlation.

**Table 4:** Brain regions exhibiting significant correlations between Stroop interference and rGMV.

No	Included gray matter areas* (number of significant voxels in left and right side of each anatomical area)	x	y	z	TFCE value	Corrected *p*-value (TFCE, FWE)	Cluster size (voxel)
1	Amygdala (L:300, R:272)/Angular gyrus (L:1500, R:536)/Calcarine Cortex (L:1629, R:1359)/Caudate (L:265, R:33)/Anterior cingulum (L:2843, R:2825)/Middle cingulum (L:4110, R:4895)/Posterior cingulum (L:477, R:265)/Cuneus (L:1249, R:1129)/Inferior frontal operculum (L:1460, R:1240)/Inferior frontal orbital area (L:2173, R:1588)/Inferior frontal triangular (L:3346, R:1529)/Middle frontal medial area (L:1682, R:2000)/Middle frontal orbital area (L:1505, R:590)/Middle frontal other areas (L:6295, R:1883)/Superior frontal medial area (L:5959, R:4901)/Superior frontal orbital area (L:1654, R:759)/Superior frontal other areas (L:3081, R:6312)/Fusiform gyrus (L:655, R:1842)/Heschl gyrus (L:420, R:263)/Hippocampus (L:260, R:447)/Insula (L:4147, R:3986)/Lingual gyrus (L:1343, R:2447)/Inferior occipital lobe (L:169, R:116)/Middle occipital lobe (L:927, R:2)/Superior occipital lobe (L:1058, R:605)/Pallidum (L:137, R:198)/Paracentral lobule (L:1011, R:938)/Parahippocampal gyrus (L:117, R:258)/Inferior parietal lobule (L:3657, R:1543)/Superior parietal lobule (L:2968, R:1020)/Postcentral gyrus (L:5990, R:3403)/Precentral gyrus (L:3275, R:3920)/Precuneus (L:4421, R:3793)/Putamen (L:762, R:1416)/Rectus gyrus (L:1896, R:1045)/Rolandic operculum (L:1983, R:2416)/Supplemental motor area (L:4094, R:4204)/Supramarginal gyrus (L:2560, R:696)/Inferior temporal gyrus (L:3488, R:1560)/Middle temporal gyrus (L:2942, R:2190)/Temporal pole (L:2838, R:769)/Superior temporal gyrus (L:2826, R:1294)/Thalamus (L:13, R:45)/Cerebellum (L:6384, R:6998)/	−10.5	−16.5	33	5168.35	0.0004	213372
2	Angular gyrus (R:107)/Middle occipital lobe (R:229)/	−15	70.5	13.5	1492.97	0.045	4
3	Middle occipital lobe (R:3)/Middle temporal gyrus (R:146)/	39	−70.5	25.5	1474.52	0.047	359
4	None	55.5	−63	15	1439.87	0.049	160

After correcting for the effects of age, sex, control task performance, and total GMV, whole-brain multiple regression analyses revealed no significant correlations between Stroop interference and rGMV.

### Relationships between performance on a complex processing speed task involving spatial cognition and rGMV

**Figure 4:**
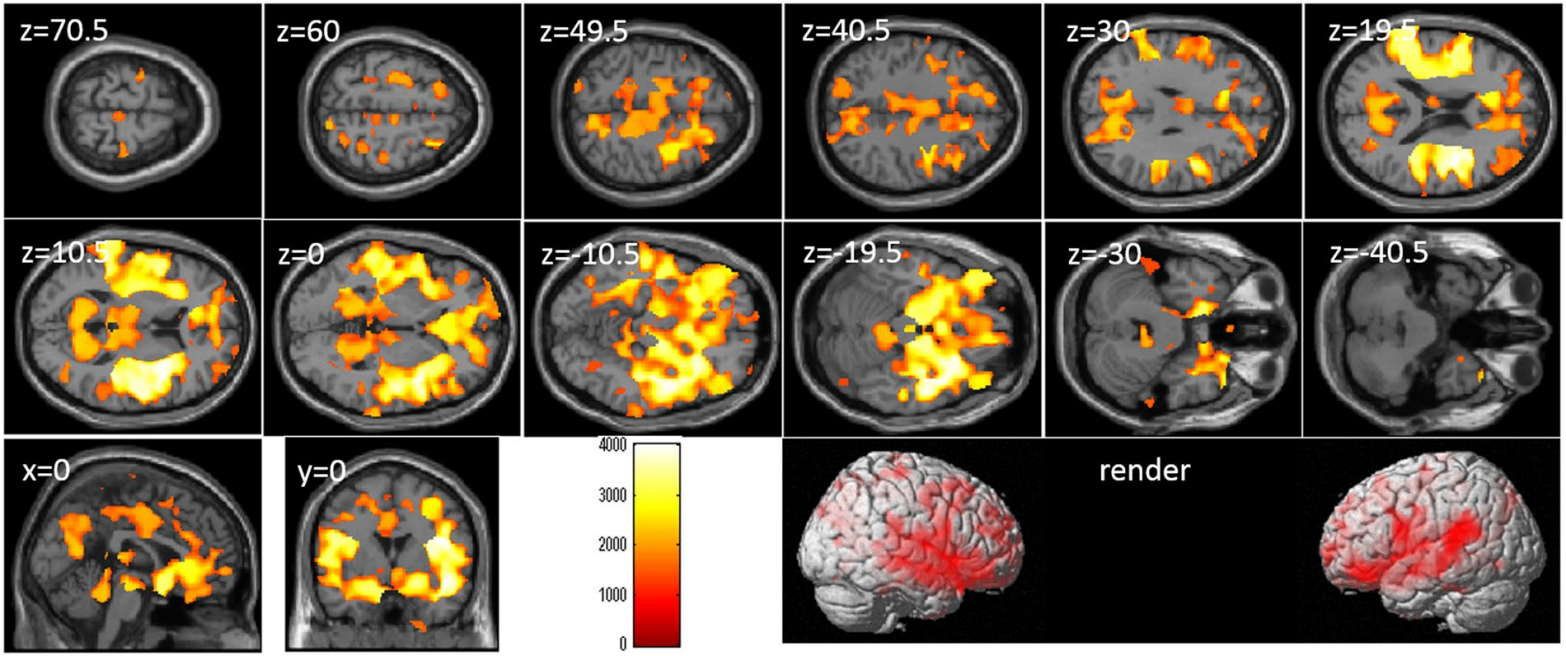
Relationships between complex processing speed involving spatial cognition and rGMV. Figures represent areas of significant positive correlation between Tanaka B-type intelligence test (TBIT) spatial factor scores and rGMV by multiple regression analysis corrected for effects of age and sex. The results shown were obtained using a threshold of TFCE, *P* < 0.05 corrected (FWE) based on 5000 permutations. In section images, the color bar represents TFCE values. Significant positive correlations were found across the whole brain.

After correcting for the effects of age and sex, whole-brain multiple regression analyses revealed significant correlations between TBIT reasoning factor score and rGMV across a wide range of brain areas (Table 5 and Fig. 4). In all brain regions of the AAL atlas, there were voxels of significant correlation.

**Table 5:** Brain regions exhibiting significant correlations between TBIT spatial factor scores and rGMV.

No	Included gray matter areas* (number of significant voxels in left and right side of each anatomical area)	x	y	z	TFCE value	Corrected *p*-value (TFCE, FWE)	Cluster size (voxel)
1	Amygdala (L:486, R:569)/Angular gyrus (L:1166, R:1293)/Calcarine Cortex (L:2850, R:2918)/Caudate (L:246, R:510)/Anterior cingulum (L:2468, R:2077)/Middle cingulum (L:3490, R:4104)/Posterior cingulum (L:994, R:723)/Cuneus (L:1194, R:1679)/Inferior frontal operculum (L:1345, R:814)/Inferior frontal orbital area (L:3824, R:2903)/Inferior frontal triangular (L:1904, R:2052)/Middle frontal medial area (L:1043, R:1673)/Middle frontal orbital area (L:1549, R:1090)/Middle frontal other areas (L:4687, R:6004)/Superior frontal medial area (L:2201, R:1075)/Superior frontal orbital area (L:1476, R:1643)/Superior frontal other areas (L:1636, R:2302)/Fusiform gyrus (L:3156, R:4708)/Heschl gyrus (L:267, R:581)/Hippocampus (L:2037, R:2247)/Insula (L:4206, R:4203)/Lingual gyrus (L:2203, R:4180)/Inferior occipital lobe (L:1014, R:1010)/Middle occipital lobe (L:1415, R:1689)/Superior occipital lobe (L:1351, R:1236)/Pallidum (L:245, R:349)/Paracentral lobule (L:884, R:1388)/Parahippocampal gyrus (L:1824, R:2500)/Inferior parietal lobule (L:1996, R:1466)/Superior parietal lobule (L:2513, R:2582)/Postcentral gyrus (L:4783, R:3828)/Precentral gyrus (L:3473, R:3615)/Precuneus (L:5978, R:6108)/Putamen (L:1477, R:1374)/Rectus gyrus (L:2062, R:1878)/Rolandic operculum (L:1290, R:2287)/Supplemental motor area (L:1255, R:1140)/Supramarginal gyrus (L:1446, R:1545)/Inferior temporal gyrus (L:2624, R:5258)/Middle temporal gyrus (L:4674, R:5813)/Temporal pole (L:1747, R:2570)/Superior temporal gyrus (L:3739, R:4351)/Thalamus (L:2144, R:2124)/Cerebellum (L:1516, R:3490)/	3	−69	46.5	5210.22	0.0002	247565

After correcting for the effects of age, sex, and total GMV, whole-brain multiple regression analyses revealed no significant correlations between TBIT reasoning factor score and rGMV.

### Relationships between performance on a complex processing speed task involving reasoning and rGMV

**Figure 5:**
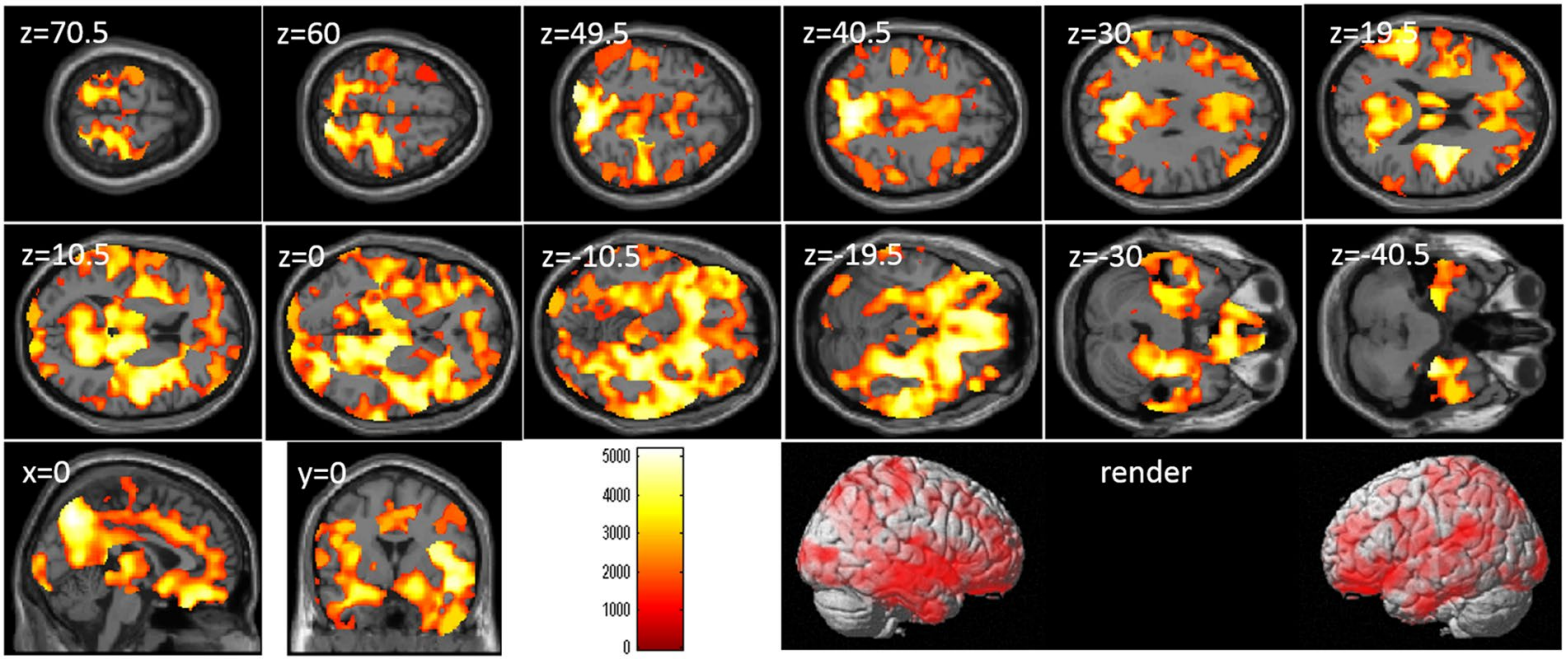
Relationships between complex processing speed involving reasoning and rGMV. Figures show areas of significant negative correlation between TBIT reasoning factor scores and rGMV by multiple regression analysis corrected for effects of age and sex. The results shown were obtained using a threshold of TFCE, *P* < 0.05 corrected (FWE) based on 5000 permutations. In section images, the color bar represents TFCE values. Significant positive correlations were found across the whole brain.

After correcting for the effects of age and sex, whole-brain multiple regression analysis revealed significant correlations between TBIT reasoning factor score and rGMV across a wide range of brain areas (Table 6 and Fig. 5). In all brain regions of the AAL atlas except the right inferior parietal lobule and right angular gyrus, there were voxels of significant correlation.

**Table 6:** Brain regions exhibiting significant correlations between TBIT reasoning factor scores and rGMV.

No	Included gray matter areas* (number of significant voxels in left and right side of each anatomical area)	x	y	z	TFCE value	Corrected *p*-value (TFCE, FWE)	Cluster size (voxel)
1	Amygdala (L:524, R:585)/Angular gyrus (L:71, R:1)/Calcarine Cortex (L:1606, R:1634)/Caudate (L:355, R:296)/Anterior cingulum (L:2498, R:1967)/Middle cingulum (L:2767, R:2727)/Posterior cingulum (L:287, R:48)/Cuneus (L:1102, R:1056)/Inferior frontal operculum (L:1760, R:1848)/Inferior frontal orbital area (L:3514, R:3234)/Inferior frontal triangular (L:833, R:1212)/Middle frontal medial area (L:1222, R:1079)/Middle frontal orbital area (L:786, R:319)/Middle frontal other areas (L:2111, R:4222)/Superior frontal medial area (L:1855, R:1101)/Superior frontal orbital area (L:631, R:558)/Superior frontal other areas (L:3509, R:2739)/Fusiform gyrus (L:581, R:571)/Heschl gyrus (L:559, R:581)/Hippocampus (L:1350, R:1775)/Insula (L:4220, R:4411)/Lingual gyrus (L:1444, R:1449)/Inferior occipital lobe (L:212, R:26)/Middle occipital lobe (L:114, R:443)/Superior occipital lobe (L:677, R:912)/Pallidum (L:24, R:128)/Paracentral lobule (L:15, R:1069)/Parahippocampal gyrus (L:1184, R:1590)/Inferior parietal lobule (L:38)/Superior parietal lobule (L:329, R:721)/Postcentral gyrus (L:1266, R:738)/Precentral gyrus (L:2476, R:2496)/Precuneus (L:2186, R:3574)/Putamen (L:416, R:1191)/Rectus gyrus (L:1382, R:1404)/Rolandic operculum (L:2267, R:2775)/Supplemental motor area (L:842, R:1360)/Supramarginal gyrus (L:1240, R:699)/Inferior temporal gyrus (L:350, R:295)/Middle temporal gyrus (L:4400, R:2672)/Temporal pole (L:1199, R:2665)/Superior temporal gyrus (L:4711, R:3970)/Thalamus (L:1455, R:1308)/Cerebellum (L:235, R:785)/	39	10.5	16.5	4020.64	0.001	155353
2	Fusiform gyrus (R:10)/Lingual gyrus (R:3)/	46.5	−76.5	−19.5	1326.29	0.050	38
3	None	−6	22.5	9	1323.46	0.050	3

After correcting for the effects of age, sex, and total GMV, whole-brain multiple regression analyses revealed no significant correlations between TBIT reasoning factor score and rGMV.

### Relationships between simple processing speed in a perceptual task and rGMV

**Figure 6:**
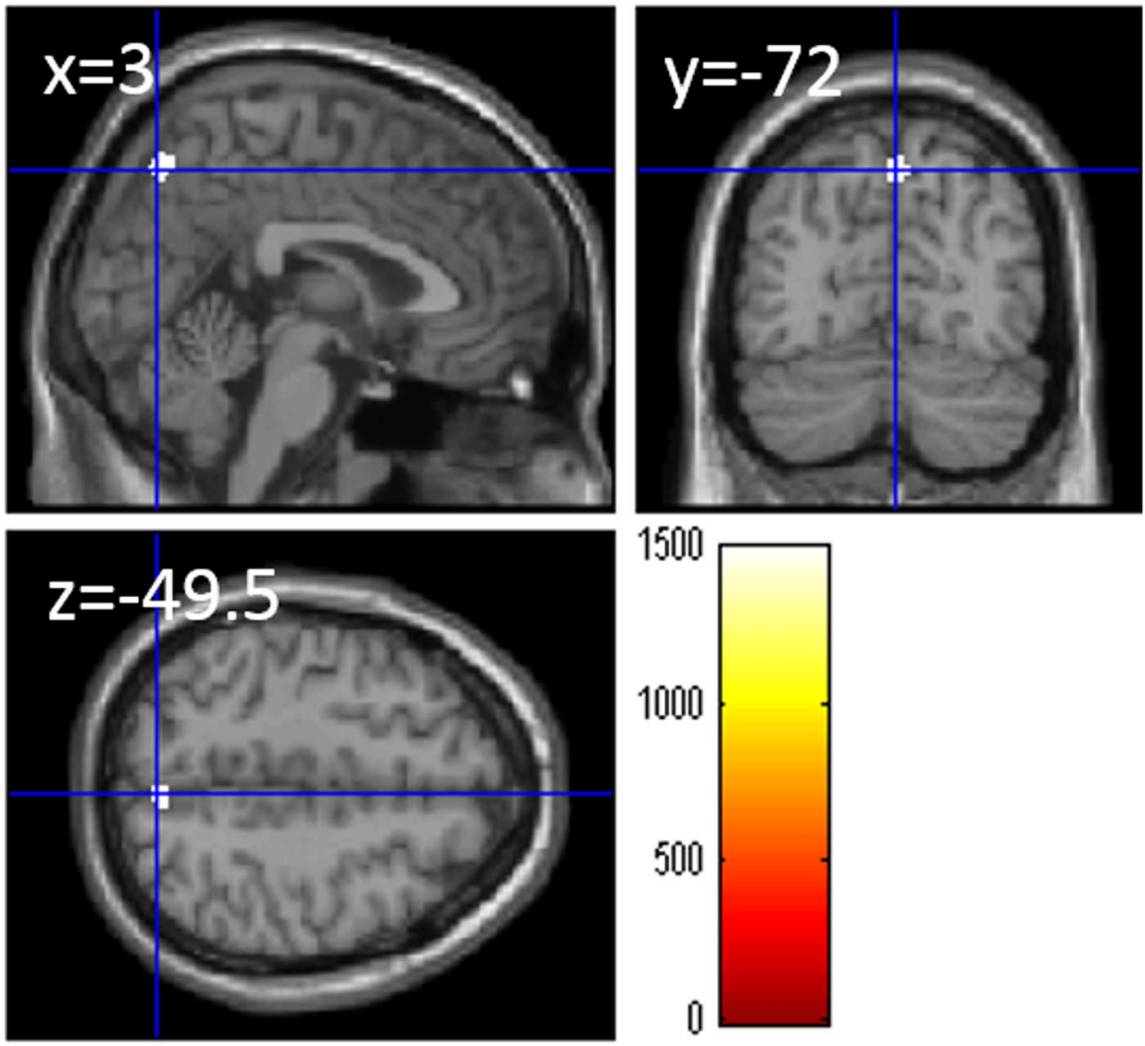
Relationships between simple processing speed and rGMV. Figures represent areas of significant positive correlation between TBIT perceptual factor scores and rGMV by multiple regression analysis corrected for effects of age and sex. The results shown were obtained using a threshold of TFCE, *P* < 0.05 corrected (FWE) based on 5000 permutations. In section images, the color bar represents TFCE values. Significant positive correlations were found in the precuneus.

After correcting for the effects of age and sex, whole-brain multiple regression analysis revealed a significant correlation between TBIT perceptual factor score and rGMV in the precuneus (*x*, *y*, *z* = 3, −72, 49.5, TFCE value = 1489.24, *P* = 0.043, corrected for multiple comparisons (FWE), 100 voxels, Fig. 6).

After correcting for the effects of age, sex, and total GMV, whole-brain multiple regression analysis revealed no significant correlations between TBIT perceptual factor score and rGMV.

### Results of effects of g factor on rGMV and the effects of ‘g-independent’ scores for each test on rGMV

**Figure 7:**
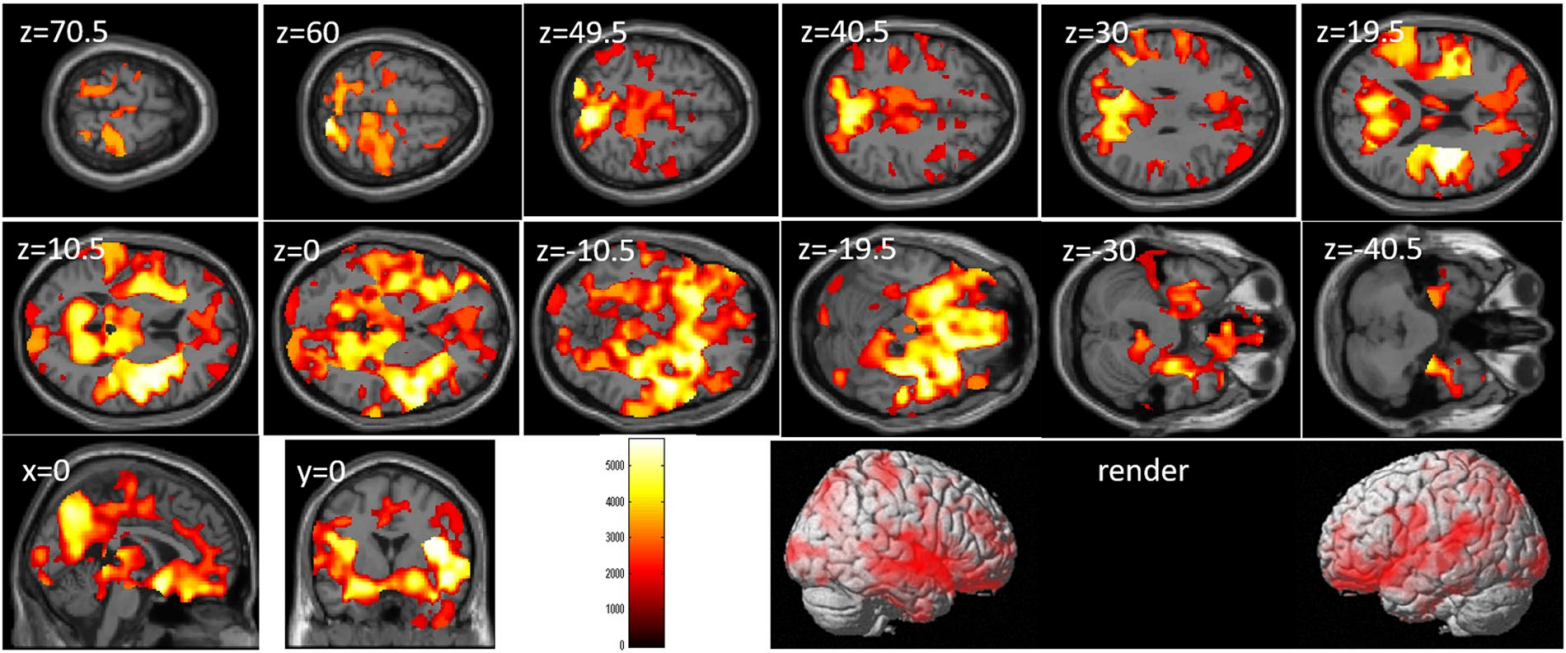
Relationships between the general intelligence factor g and rGMV. Figures represent areas of significant positive correlation between the general intelligence factor g calculated from the five CCA measures in this study (excluding Stroop interference) and rGMV by multiple regression analysis corrected for the effects of age and sex. The results shown were obtained using a threshold of TFCE, *P* < 0.05 corrected (FWE) based on 5000 permutations. In section images, the color bar represents TFCE values. Significant positive correlations were found across the whole brain.

After correcting for the effects of age and sex, a whole-brain multiple regression analysis revealed significant correlations between g factor calculated from five tasks (except stroop interference) in this study and rGMV across a wide range of brain areas (Supplemental Table 3 and Fig. 7). In all brain regions of AAL atlas, there were voxels of significant correlation.

**Figure 8:**
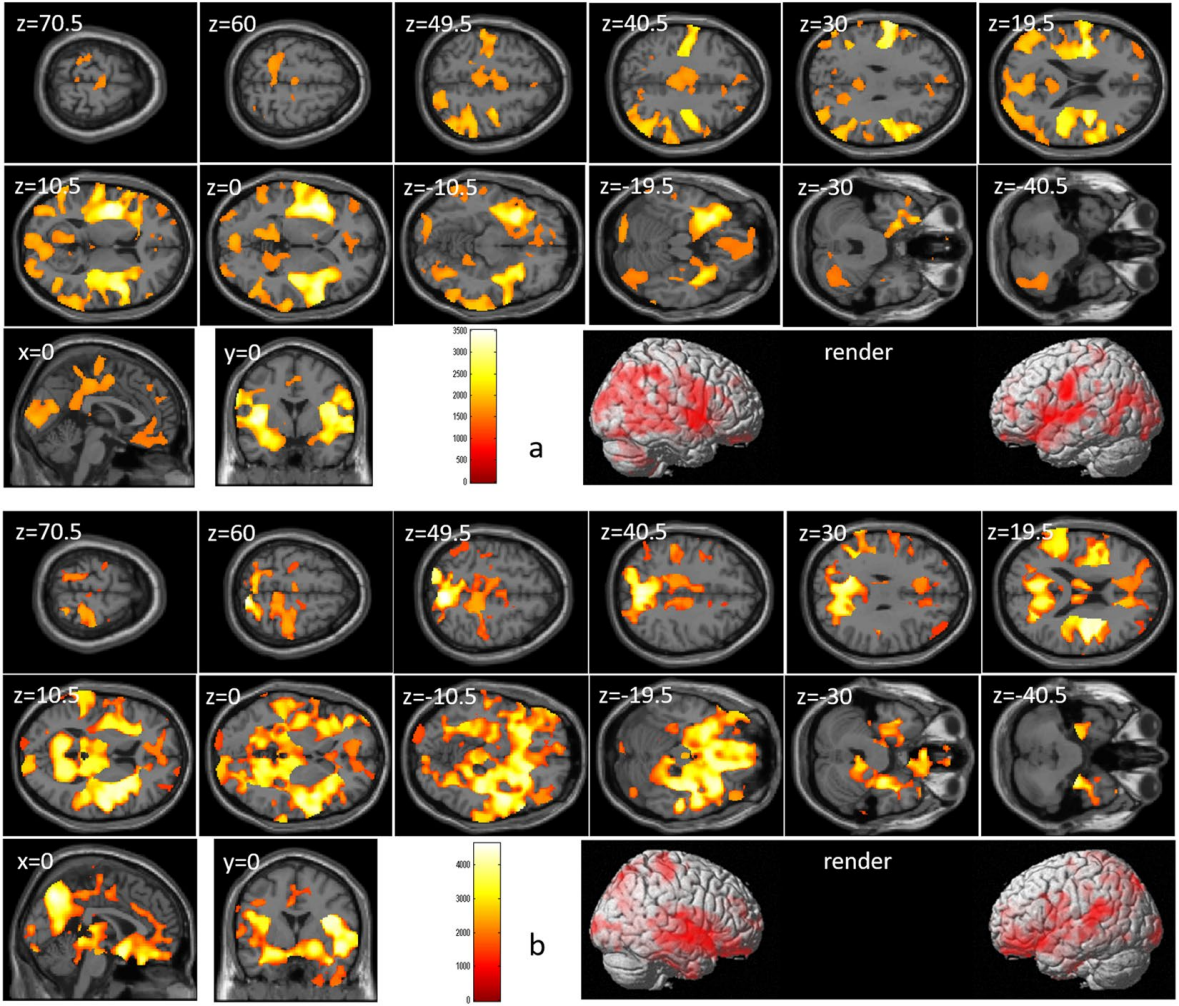
Relationships between rGMV and the “g-independent” digit span score as well as the general intelligence factor g. Figures represent areas of significant positive correlation of rGMV with the (**a**) “g-independent” digit span score and (**b**) general intelligence factor calculated from the four CCA measures in this study (excluding Stroop interference and digit span) by multiple regression analysis including age, sex, and these two CCA measures as independent variables. The results shown were obtained using a threshold of TFCE, *P* < 0.05 corrected (FWE) based on 5000 permutations. In section images, the color bar represents TFCE values. Significant positive correlations were found for the “g-independent” digit span score and general intelligence factor in most anatomical areas of the whole brain (with the general exception of the dorsal part of the brain, where significant voxels were scarce).

As for g-independent digit span score, after correcting for the effects of age, sex, and g factor calculated from the other four tasks (except stroop interference), a whole-brain multiple regression analysis revealed significant correlations between g-independent digit span score and rGMV across a wide range of brain areas (Supplemental Table 4 and Fig. 8). In all brain regions of AAL atlas except the orbital part of the right middle frontal gyrus, right pallidum, and left thalamus, there were voxels of significant correlation. In this same multiple regression analysis, g factor calculated from the other four tasks (except stroop interference) showed significant positive correlations with rGMV in all brain regions except the right angular gyrus (Supplemental Table 5 and Fig. 8b). However, in both of correlations as can be seen in Fig. 8a,b, there were less significant voxels in the dorsal parts of the brain.

**Figure 9:**
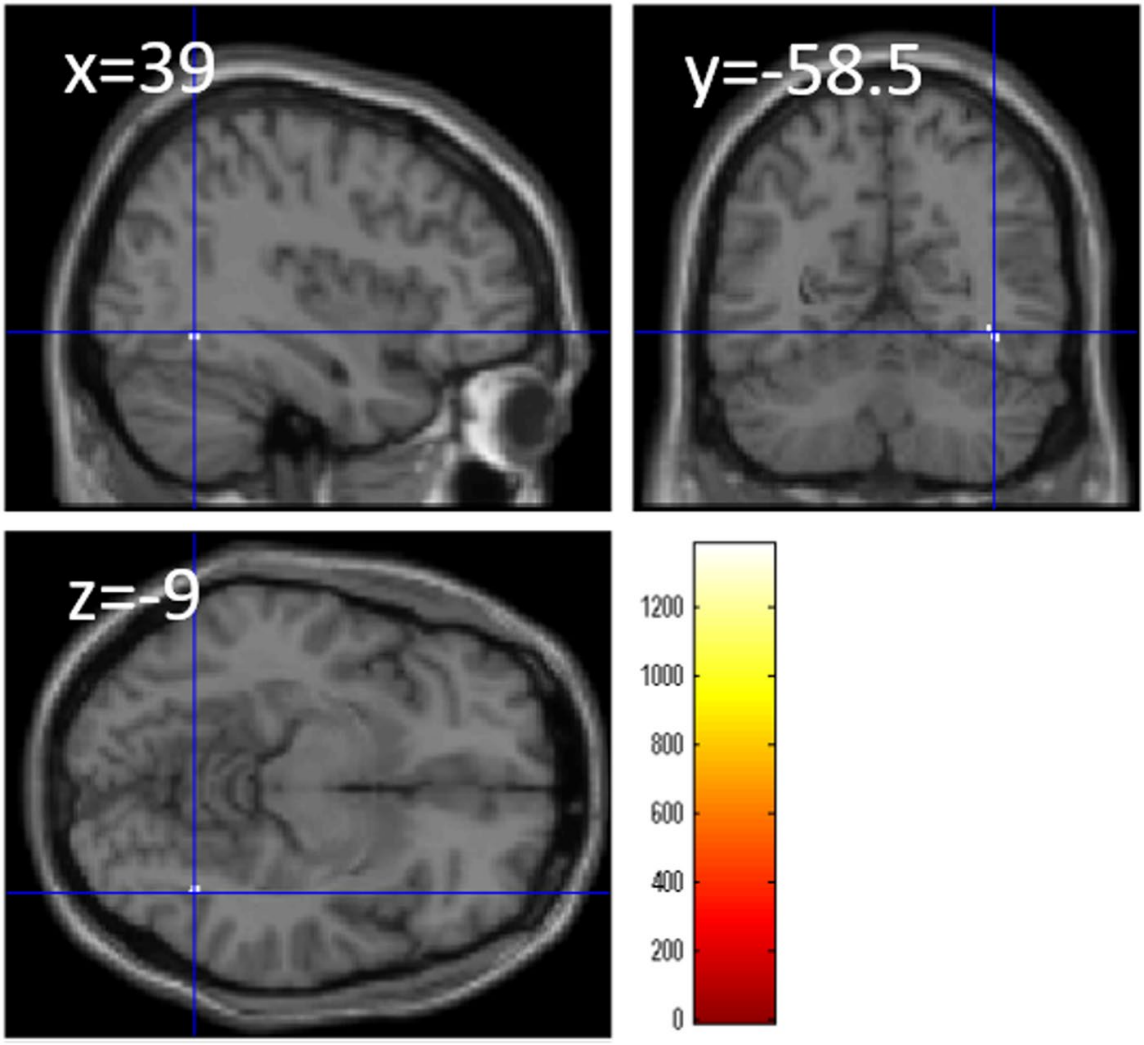
Relationships between the “g-independent” complex processing speed involving spatial cognition and rGMV. Figures represent areas of significant negative correlation between the TBIT spatial relation factor score independent of the general intelligence factor g and rGMV by multiple regression analysis corrected for the effects of age, sex, and general intelligence factor (as calculated from the other CCA tasks excluding Stroop interference). The results shown were obtained using a threshold of TFCE, *P* < 0.05 corrected (FWE) based on 5000 permutations. In section images, the color bar represents TFCE values. Significant positive correlations were found in the right fusiform gyrus.

As for g-independent spatial factor of TBIT, after correcting for the effects of age, sex, and g factor calculated from the other four tasks (except stroop interference), a whole-brain multiple regression analyses revealed a significant correlation between g-independent spatial factor of TBIT and rGMV in the right fusiform gyrus (Fig. 9, x, y, z = 39, −58.5, −9, TFCE value = 1376.41, 16 voxels, corrected for multiple comparisons).

For these 3 scores, the beta values of the multiple regression analyses of associations between scores and rGMV for each anatomical area are presented in Supplemental Table 6, and the r value of simple regression analyses in males and females are provided in Supplemental Table 7.

As for, g-independent RAPM score (non-verbal reasoning), g-independent reasoning factor of TBIT (number reasoning), g-independent perceptual factor of TBIT (simple processing speed), there were no significant correlations. However, there was a tendency of negative correlation between rGMV and g-independent perceptual factor of TBIT in the frontal lobe.

## Discussion

This study investigated the associations between diverse CCAs and rGMV. We employed a large sample size, the newest pre-processing techniques, and robust permutation-based statistical techniques. Consistent with our initial hypothesis, among five complex CCAs examined, significant associations between performance on four and greater rGMV were found across widespread brain areas. Furthermore, the beta values of the strongest associations between rGMV in the predefined anatomical areas and CCA scores were only around 0.15 in the strongest case. Thus, we consider these associations very weak, again consistent with our hypothesis. These results cannot be attributed to the fact that we did not use the general intelligence factor g in these analyses as correlations between rGMV and the factor that consists from 5 measures in this study showed the same pattern (Fig. 7). The effect of sex cannot be ignored, threfore, r values of simple regression analyses were not used to indicate strength of the associations in the whole sample in this study. As per some previous studies^45–48^, we used beta values to indicate the strength of the associations. However, the beta values and means of r values of the male and female samples were comparable (Supplemental Tables 2 and 7).

The first important implication of the present results is that they provide a comprehensive explanation for the diverse findings and lack of replicability in previous studies on the relationships between CCA and cortical structure. The present findings of generally weak associations between rGMV and CCAs (Supplemental Table 1) were obtained using predefined anatomical masks and a huge sample size. Thus, these findings are relatively free from the overfitting and consequent overestimation of effect size in previous whole-brain analyses^49^ as well as the low statistical power of small sample size studies^50^. We thus consider these effect sizes close to a real strength. The present weak effects are congruent with the notion that previous studies lacked replicability (as outlined by ref. 13) not only because of low statistical power (i.e., to obtain significant effects in whole-brain analyses after corrections for multiple comparisons) but also because the real effects sizes of rGMV on CCA performance are truly low. In addition, the widespread correlations found in this study are congruent with the notion that the significant regional structural correlations with CCAs reported in previous studies (as reviewed by Jung *et al*.^9^) are real.

Several related studies have been performed and compared with these studies, by using a huge sample size, we could show comprehensive pictures of the associations between CCA and rGMV. As described, numerous studies have investigated gray matter structural correlates of psychometric intelligence (for review, see ref. 9), although relatively few have investigated gray matter structural correlates of certain CCAs such as WMC^11^ and the attention/inhibition component of executive function^12^. However, findings have been highly inconsistent. Indeed, different studies have shown correlations in different areas, and no single specific correlation was found in more than half of the studies. Further, almost all brain areas analyzed showed some level of correlation with psychometric intelligence, in at least some studies^13^. A recent meta-analysis^10^ concluded that there were positive associations between regional gray matter structure and psychometric intelligence in several confined areas. However, the meta-analysis included only 457 subjects from multiple sources; therefore, their findings could be underpowered. Other researchers performed studies that are similar to our present study. Colom *et al*.^11^ investigated the associations between rGMV and fluid, crystallized, and spatial intelligence, working memory, attention, and processing speed in 104 young adults. They showed both distinct and overlapping anatomical correlates of these cognitive functions. In hindsight, their studies were also underpowered. Karama *et al*.^42^ investigated the relationships between cortical thickness and both general intelligence and a wide range of specific CCAs in 207 children. They successfully showed positive correlations between widespread cortical thickness and g (which contributes to success in many cognitive tests)^4^ as well as more selective correlation patterns between cortical thickness and specific CCAs. However, after regressing out g, these selective correlations between CCAs and cortical thickness disappeared. They suggested that many CCAs show widespread correlations (like g) only when using a very lenient threshold, but their claims were not verified statistically. The present study recruited a huge sample within a limited age range for examination at a single site. Although the different cognitive domains examined were represented by a single measure (one score), which may reduce statistical power, the unified huge sample provides a comprehensive picture of the associations between rGMV and a wide range of CCAs. Namely, a wide range of CCAs showed weak correlations in almost all brain areas. This strongly suggests that previous analyses with small sample sizes are likely to lead to significant correlations only in certain areas among larger regions with significant correlations. Another important advance is that we successfully identified rGMV correlations with the “g-independent” digit span score and “g-independent” spatial relation factor of TBIT. These results suggest that associations between cortical structures and cognitive performance cannot be explained merely by associations between cortical structures and general intelligence. This is in contrast to previous studies that failed to find effects of individual CCAs that were independent from general intelligence.

The reasons why individual CCAs are associated with rGMV over widespread areas rather than confined regions are not clear, but we suggest a few possibilities. In functional imaging analyses, brain regions activated during cognitive tasks are clearly segregated from those deactivated^51^. But in the present study, rGMV of widespread rather unspecific areas were correlated with diverse CCAs. One possible explanation is provided by recent findings on brain structure by^52^. In that study, rGMV in one area was strongly correlated with rGMV in the contralateral area and contingent areas regardless of the network to which these contingent areas belong^52^. Also, the rGMV of each area is strongly explained by global effects^19^. Thus, even when only a few areas or networks are functionally associated with a given CCA, multiple contingent areas are associated with the CCA tested. The other possibility is that many of the major environmental factors affecting the development of human psychometric intelligence are associated with differences in global brain development or damage (e.g., prenatal exposure to alcohol or tobacco, preterm status, low socioeconomic status, parental age, and anorexia)^53^. Thus, through effects of these factors, associations may be formed between CCA and rGMV of widespread brain areas.

The second important implication is that the present results suggest one fundamental limitation of rGMV analysis to map brain areas involved in CCA. The important finding of this study is that many CCAs are weakly associated with rGMV of widespread areas across the whole brain (e.g., betas were generally around 0.1 in most of the predefined anatomical areas, Supplemental Table 1). Except for the right insula in the case of verbal WM (digit span), no significant associations were found between CCAs and rGMV after regressing out the effects of total gray matter volume, despite the large sample size. When the associations between rGMV and “g-independent” specific cognitive test scores were investigated, only the “g-independent” digit span and “g-independent” spatial relation factor of TBIT showed significant correlations despite the large sample size. Furthermore, patterns of associations between rGMV and the “g-independent” digit span score were similar to those of the general intelligence factor g, and associations were widespread in numerous areas (Fig. 8a,b). Thus, if the fundamental purpose of such analyses is to identify specific brain regions involved in specific CCAs, our results suggest that this goal cannot be easily achieved using these kinds of analyses. In the similar way, it may cast doubt on the authenticity of the notion that our CCAs can be estimated by just looking at the brain structures at least in the normal young adult.

The third important implication is about several hundred subjects may be required for these kinds of analyses under the traditional statistical criteria. In this study, we could induce the confidence interval rate of the beta coefficients in each ROI. The simple correlation coefficients in each sex are similar to these values. Even in the case of significance, it is not much larger than 0.1. According to the statistical power analysis, when we have to detect the correlation of r = 0.1 by the probability of 80% under the threshold of p < 0.05, about 783 subjects are required. In imaging analyses, due to the freedom of choice of the methods of correction for multiple comparisons, things are more complicated. But the present results and these statistical power analyses tell that the large sample size is required to effectively depict the comprehensive picture of the associations between cognitive differences and structures in this kind of sample.

Some might believe progress in science might be more likely achieved if some specific, theoretically founded research questions are tested in carefully selected sample of participants and overpowered studies are associated with the problem that very small and perhaps negligible effects reach significance due to the huge sample size. But to make a counterargument for these beliefs, we just have to look at history of the study of polymorphisms which generated numerous false positive as a result of application of these kinds of beliefs to detect the small effects. What we showed was actually, the effect size of the associations between brain structural properties and basic cognitive functions are small. Whether the studies with huge sample power detect even the negligible effects or not, the problem is the effects to be found are small from the beginning.

This study did find two significant associations between a specific CCA and a defined cortical region, a positive correlation between rGMV in the insula and verbal WM performance after regressing out effects of total GMV, and a positive correlation between rGMV in the precuneus and simple processing speed. We also found an association between “g-independent” spatial complex cognitive speed and rGMV in the right fusiform gyrus. These findings are not central to our hypothesis, but we discuss implications here. The insula is consistently activated during working memory tasks^51^. However, the insula is involved in many functions^54^ and so is one region where reverse inference is difficult^55^. Nonetheless, if we have to speculate on the meaning of this finding, cognitive processes such as inhibition that are ascribed to this area^54^ and that play key roles in WM^56^ may account for the association. Similarly, the precuneus is involved in many functions^57^, but the association of this area with visuospatial processing suggests greater involvement given the non-verbal nature of the tasks employed. Finally, the fusiform gyrus is known to be involved in number recognition^58^, and the development of this cortical area is affected by socioeconomic status^59^. Further, there are strong associations between arithmetic and spatial cognition^60^. Thus, one plausible explanation for the associations of “g-independent” spatial complex processing speed and rGMV is the association between spatial ability and numerical problems^58^. However, the fusiform gyrus is associated with the recognition of numerous forms of information, so this speculation must be confirmed in future studies. In addition, it is now known that rGMV mixes different sources of anatomical variability, including cortical thickness, cortex gyrification, cortical surface area^61^, but due to so many analyses, we could only investigate one gray matter structural indices. Whether other gray matter indices show the similar patterns of results in this study have to be investigated in future studies.

In this study, despite the significant associations between rGMV and verbal WM, the attention/inhibition component of executive function, and complex processing speeds, RAPM score was not significantly correlated with rGMV despite the strong association between RAPM score and general intelligence^3^. As described in the Results section, positive trends were found in areas identified in a previous study using similar methods. Thus, the present results are far from null correlations, but the interpretation is difficult at present. The lack of significance involving RAPM score (compared to other significant analyses) may well be explained by random statistical variation. However, if we are forced to present one possibility of the reason of lack of significant correlations, previously we suggested cognitive functions requiring faster processing or greater amount of information and faster cognitive processes may show positive correlation with rGMV^26^. Compared with other tasks that are employed in this study, RAPM may require these processes less. Nonetheless, due to lack of significant correlations in the analysis of RAPM, we cannot not be sure if all or most of the CCAs, show correlations with widespread rGMV.

The present findings of weak associations between cognitive performance and rGMV may appear incongruent with a previous study^62^ of 672 adults reporting that three types of brain structural imaging data can account for 18–21% of the variance in intelligence. However, that study dealt only with older adults (average age of 72.49 years), and around this age, humans can exhibit brain atrophy and associated cognitive decline, both of which vary substantially among individuals^63, 64^. In contrast, we examined a young cohort within a narrow age range (thereby eliminating these sources of variability).

Our study has a limitation common to previous studies investigating the associations between neural mechanisms and individual cognitive differences; our study population is restricted to young healthy subjects (18–27 years old) with higher education levels^40, 65, 66^. One previous study found that the direction (positive/negative) of the associations between psychometric intelligence and brain cortical structures changes with age^67^. However, this study suggests the importance of focusing on one age range to investigate the associations between CCA and cortical structures. Nonetheless, whether the present findings hold true for other age groups requires investigation.

In conclusion, our study showed that different facets of CCA [verbal working memory capacity (WMC), the attention/inhibition component of executive function, and complex processing speeds involving spatial cognition and those involving reasoning] are associated with rGMV across widespread gray matter areas. The strengths of individual associations are rather weak and highly distributed. A well-referred meta-analysis concluded that total brain volume and psychometric intelligence are weakly but robustly related^18^. Individual studies have found trends or significant correlations between CCAs and diverse gray matter structures^9^ but are sometimes regarded as unreplicable^13^. Our study utilized the newest preprocessing method of VBM. Through rigorous preprocessing and statistical techniques and by using a large sample, our findings suggest that multiple CCAs are indeed associated with highly distributed anatomical regions with only weak correlations to specific regions. This lack of strong correlation with any one area, combined with the stringent corrections of multiple comparisons, might lead to different and diverse findings in the field.

## Electronic supplementary material

Supplementary online material
