# Supplementary material for: Global associations between regional gray matter volume and diverse complex cognitive functions: evidence from a large sample study
Source: Sci Rep. 2017 Aug 30;7:10014. doi: 10.1038/s41598-017-10104-8 (PMC5577279; doi:10.1038/s41598-017-10104-8)
Supplement: Supplementary file 1 — Supplementary online material [file 41598_2017_10104_MOESM1_ESM.pdf]

Supplemental online material

Global associations between regional gray matter volume and diverse complex cognitive functions: evidence from a large sample study

Hikaru Takeuchi[a], Yasuyuki Taki[a,b,c], Rui Nouchi[d,e,f], Ryoichi Yokoyama[g], Yuka Kotozaki[h], Seishu Nakagawa[i,j], Atsushi Sekiguchi[k], Kunio Iizuka[l], Yuki Yamamoto[i], Sugiko Hanawa[i], Tsuyoshi Araki[f], Carlos Makoto Miyauchi[m], Takamitsu Shinada[i], Kohei Sakaki[i], Yuko Sassa[a], Takayuki Nozawa[n], Shigeyuki Ikeda[n], Susumu Yokota[a], Magistro Daniele[o], Ryuta Kawashima[a,f,i]

[a]*Division of Developmental Cognitive Neuroscience, Institute of Development, Aging and Cancer, Tohoku University, Sendai, Japan*

[b]*Division of Medical Neuroimaging Analysis, Department of Community Medical Supports, Tohoku Medical Megabank Organization, Tohoku University, Sendai, Japan*

[c]*Department of Radiology and Nuclear Medicine, Institute of Development, Aging and Cancer, Tohoku University, Sendai, Japan*

[d]*Creative Interdisciplinary Research Division, Frontier Research Institute for Interdisciplinary Science, Tohoku University, Sendai, Japan*

[e]*Human and Social Response Research Division, International Research Institute of Disaster Science, Tohoku University, Sendai, Japan*

[f]*Smart Ageing International Research Center, Institute of Development, Aging and Cancer, Tohoku University, Sendai, Japan*

[g] *School of Medicine, Kobe University, Kobe, Japan*

[h]*Division of Clinical research, Medical-Industry Translational Research Center, Fukushima Medical University School of Medicine, Fukushima, Japan*

[i]*Department of Functional Brain Imaging, Institute of Development, Aging and Cancer, Tohoku University, Sendai, Japan*

[j]*Department of Psychiatry, Tohoku Pharmaceutical University, Sendai, Japan*

[k]*Department of Adult Mental Health, National Institute of Mental Health, National Center of Neurology and Psychiatry, Kodaira, Tokyo, Japan*

[l]*Department of Psychiatry, Tohoku University Graduate School of Medicine, Sendai, Japan*

[m]*Graduate School of Arts and Sciences, Department of General Systems Studies, The University of Tokyo, Tokyo, Japan*

[n]*Department of Ubiquitous Sensing, Institute of Development, Aging and Cancer, Tohoku University, Sendai, Japan*

[o]*School of Electronic, Electrical and Systems Engineering, Loughborough University, England*

Corresponding author:

Hikaru Takeuchi

Division of Developmental Cognitive Neuroscience, IDAC, Tohoku University

4-1 Seiryo-cho, Aoba-ku, Sendai 980-8575, Japan

Tel/Fax: +81-22-717-7988

E-mail: takehi@idac.tohoku.ac.jp

Running title: Complex cognitive functions and brain structure

Keywords:

Regional gray matter volume; working memory capacity; attention; complex processing speed; non-verbal reasoning; voxel-based morphometry

Supplemental Methods.

Assessment of psychometric measures of non-verbal reasoning. The methods for psychometric measurement of non-verbal reasoning were reproduced from our previous study [1]. Raven's Advanced Progressive Matrix (RAPM) [2] is a non-verbal reasoning task shown to be well correlated with general intelligence. Thus, we used RAPM to assess non-verbal reasoning abilities in this study. For more details of how RAPM was performed, see our previous works [3, 4].

Assessment of verbal WMC. Computerized forward and backward digit span tests were used to assess verbal WMC. The methods for this measure are described in our previous study [5]. Subjects were asked to view a progressively increasing number of random digits visually presented one-digit per second on a computer screen. They were then asked to repeat the sequence in the presented order (digit-span forward) or in the reverse order (digit-span backward) by pressing numbered buttons on the screen, starting from two digits. Three sequences were given at each level until the participants responded incorrectly to all three sequences, at which point the task was ended. The total score on this test is equal to the sum of how many times digits in the digit span are correctly repeated forward and backward.

Assessment of attention/inhibition component of executive functions. We used Hakoda's version of the Stroop task [6] to measure Stroop interference, which represents the attention/inhibition component of executive function. The measurement methods are described in detail in our previous study [5]. Unlike the oral naming-type Stroop task, in the matching-type stroop task (writing) subjects choose and write down as many appropriate answers as possible from five options. This type of task enables the measurement of subjects' performance correctly. We used a control Color-Word task and a Stroop task. The Stroop interference rate is calculated as follows:

Stroop interference = (correct answers on the Color-Word task − correct answers on the Stroop task)/(correct answers on the Color-Word task) × 100

In the Color-Word task, the leftmost column is painted with a color, and five other columns contain color names. The subjects have to check the column with the word corresponding to the name of the color painted in the leftmost column. In the Stroop task, a color name is printed in another color (e.g., "blue" is printed green) in the leftmost column and five other columns contain color names. The subjects have to check the column with the word corresponding to the name of the color in which the word in the leftmost column is printed [for the scheme, see our previous study [7]]. During each session, the subjects were instructed to complete as many trials as possible in 1 min.

Assessment of complex and simple processing speed. The Tanaka B-type intelligence test [8] Type 3B, which is for examinees in their 3rd year of junior high school and older, was used in this study. A description of this test can be found in our previous study [9]. This test is a nonverbal mass intelligence test which does not include story problems but uses figures, single numbers, and letters as stimuli. In all subtests, subjects had to complete as many problems as possible within a certain time (a few minutes).

This test consist of a maze test (subjects had to trace a maze with a pencil from start to finish), counting cubes (subjects had to count the number of cubes piled up in three-dimensional arrays), a displacement task (figures and numbers; subjects had to substitute a figure [9 figures] with a number [1 to 9] according to a model chart), identification vs. same-different judgments (Japanese kana characters; subjects had to judge whether a pair of meaningless Japanese strings were the same), filling in a sequence of numbers (subjects had to fill in the blanks of a number sequence with suitable numbers according to the rules of the number arrangement), marking figures (subjects had to select forms which were identical to three samples from a series [sequence] of eight different forms), and filling in figures (subjects had to complete uncompleted figures so that the

uncompleted figures were the same as the sample figures when rotated). Tasks were performed in this order.

The test consists of three sub-factors: perceptual, spatial, and reasoning. The perceptual factor score consists of the sum of scores on typical simple PS tasks: a displacement task, identification vs. same-different judgments, and marking figures. Thus, the score for the perceptual factor was treated as simple PS in this study. The spatial factor score consists of scores on a maze test, counting cubes, and filling in figures. The reasoning factor score consists of the score on a task involving filling in a sequence of numbers. The tasks for these latter two factors require relatively more complex cognitive judgments so the scores were treated as indices of complex PS.

Supplemental Table 1. Beta coefficients (95% confidence intervals) from multiple regression analyses of associations between cognitive scores and rGMV for each anatomical ROI

	RAPM	digit span	Stroop interference	TBIT spatial factors	TBIT reasoning factors	TBIT perceptual factors
Total gray matter volume	0.032(-0.013-0.076)	0.101(0.055-0.146)****	-0.08(-0.126--0.034)****	0.088(0.039-0.137)****	0.074(0.024-0.123)***	0.032(-0.016-0.079)
Amygdala L	0.046(0.001-0.092)**	0.086(0.04-0.133)****	-0.051(-0.098--0.005)**	0.075(0.025-0.124)***	0.082(0.031-0.132)***	0.036(-0.013-0.084)
Amygdala R	0.051(0.006-0.097)**	0.064(0.017-0.11)***	-0.043(-0.09-0.004)*	0.065(0.015-0.114)**	0.075(0.024-0.125)***	0.035(-0.013-0.084)
Angular gyrus L	0.03(-0.022-0.081)	0.102(0.05-0.155)****	-0.068(-0.121--0.016)**	0.068(0.011-0.124)**	0.043(-0.014-0.101)	0.038(-0.017-0.093)
Angular gyrus R	0.007(-0.044-0.057)	0.094(0.043-0.146)****	-0.053(-0.104--0.002)**	0.059(0.004-0.114)**	0.008(-0.048-0.064)	-0.004(-0.058-0.05)
Calcarine Cortex L	0.052(0.002-0.102)**	0.109(0.058-0.16)****	-0.052(-0.103--0.001)**	0.076(0.021-0.13)***	0.059(0.004-0.115)**	0.04(-0.014-0.093)
Calcarine Cortex R	0.069(0.019-0.119)***	0.103(0.052-0.154)****	-0.049(-0.1-0.002)*	0.09(0.036-0.145)***	0.067(0.012-0.123)**	0.035(-0.018-0.089)
Caudate L	-0.003(-0.054-0.047)	0.043(-0.009-0.095)	-0.027(-0.079-0.025)	0.011(-0.044-0.067)	0.014(-0.042-0.071)	-0.019(-0.073-0.035)
Caudate R	0.001(-0.05-0.052)	0.043(-0.01-0.095)	-0.023(-0.075-0.029)	0.019(-0.037-0.074)	0.02(-0.037-0.076)	-0.004(-0.058-0.051)
Anterior cingulum L	0.036(-0.015-0.086)	0.088(0.037-0.14)****	-0.086(-0.138--0.035)***	0.076(0.021-0.131)***	0.081(0.025-0.137)***	0.026(-0.028-0.08)
Anterior cingulum R	0.021(-0.029-0.07)	0.065(0.014-0.116)**	-0.085(-0.136--0.034)***	0.069(0.015-0.124)**	0.069(0.013-0.124)**	0.019(-0.035-0.072)

Middle cingulum L	0.028(-0.021-0.077)	0.097(0.046-0.148)****	-0.103(-0.153--0.053)****	0.074(0.02-0.128)***	0.075(0.02-0.13)***	0.026(-0.027-0.079)
Middle cingulum R	0.031(-0.018-0.08)	0.083(0.033-0.134)***	-0.105(-0.155--0.055)****	0.077(0.023-0.131)***	0.065(0.01-0.119)**	0.019(-0.034-0.071)
Posterior cingulum L	0.026(-0.023-0.075)	0.074(0.023-0.124)***	-0.053(-0.103--0.002)**	0.085(0.031-0.139)***	0.06(0.005-0.114)**	0.052(0-0.105)*
Posterior cingulum R	0.02(-0.029-0.069)	0.075(0.025-0.125)***	-0.049(-0.099-0.001)*	0.069(0.015-0.123)**	0.03(-0.024-0.085)	0.019(-0.034-0.071)
Cuneus L	0.039(-0.011-0.089)	0.086(0.035-0.138)****	-0.069(-0.12--0.018)***	0.062(0.008-0.117)**	0.062(0.006-0.118)**	0.028(-0.026-0.081)
Cuneus R	0.038(-0.012-0.088)	0.109(0.059-0.16)****	-0.064(-0.115--0.014)**	0.078(0.024-0.133)***	0.077(0.022-0.133)***	0.044(-0.009-0.097)
Inferior frontal operculum L	0.013(-0.039-0.065)	0.103(0.049-0.156)****	-0.079(-0.132--0.025)***	0.078(0.021-0.136)***	0.109(0.051-0.167)****	0.038(-0.018-0.094)
Inferior frontal operculum R	0.023(-0.029-0.074)	0.1(0.048-0.153)****	-0.046(-0.099-0.007)*	0.051(-0.005-0.107)*	0.085(0.028-0.142)***	0.026(-0.029-0.081)
Inferior frontal orbital area L	0.03(-0.019-0.078)	0.087(0.038-0.137)****	-0.061(-0.111--0.012)**	0.088(0.035-0.141)***	0.092(0.038-0.146)****	0.011(-0.041-0.063)
Inferior frontal orbital area R	0.029(-0.02-0.078)	0.072(0.022-0.123)***	-0.057(-0.107--0.007)**	0.076(0.023-0.13)***	0.096(0.042-0.15)****	0.008(-0.045-0.06)
Inferior frontal triangular L	-0.006(-0.056-0.045)	0.081(0.029-0.133)***	-0.069(-0.12--0.017)***	0.056(0.001-0.112)**	0.047(-0.01-0.103)	0.009(-0.045-0.063)
Inferior frontal triangular R	-0.009(-0.059-0.041)	0.066(0.014-0.117)**	-0.054(-0.105--0.003)**	0.067(0.012-0.121)**	0.062(0.006-0.117)**	-0.009(-0.062-0.045)

Middle frontal other areas L	-0.011(-0.061-0.04)	0.093(0.042-0.145)****	-0.087(-0.138--0.036)****	0.073(0.018-0.127)***	0.066(0.01-0.122)**	0.021(-0.033-0.075)
Middle frontal other areas R	0.005(-0.045-0.055)	0.083(0.032-0.134)***	-0.054(-0.105--0.003)**	0.082(0.027-0.136)***	0.081(0.026-0.136)***	0.02(-0.033-0.073)
Middle frontal medial area L	0.04(-0.01-0.09)	0.077(0.026-0.128)***	-0.081(-0.132--0.03)***	0.054(-0.001-0.109)*	0.073(0.018-0.129)***	0.01(-0.044-0.063)
Middle frontal medial area R	0.03(-0.019-0.079)	0.076(0.025-0.126)***	-0.075(-0.125--0.024)***	0.065(0.011-0.119)**	0.062(0.007-0.117)**	0.006(-0.047-0.058)
Middle frontal orbital area L	0.017(-0.032-0.066)	0.07(0.02-0.12)***	-0.069(-0.119--0.019)***	0.069(0.016-0.123)**	0.06(0.005-0.114)**	0.012(-0.04-0.064)
Middle frontal orbital area R	0.047(-0.003-0.097)*	0.048(-0.003-0.099)*	-0.047(-0.098-0.004)*	0.054(0-0.109)*	0.039(-0.017-0.094)	-0.029(-0.082-0.025)
Superior frontal other areas L	-0.026(-0.077-0.024)	0.083(0.031-0.136)***	-0.064(-0.117--0.012)**	0.047(-0.009-0.102)	0.078(0.022-0.135)***	0.025(-0.03-0.079)
Superior frontal other areas R	-0.001(-0.051-0.05)	0.08(0.028-0.131)***	-0.09(-0.142--0.039)****	0.058(0.003-0.113)**	0.074(0.018-0.13)***	0.025(-0.028-0.079)
Superior frontal medial area L	-0.009(-0.058-0.041)	0.078(0.027-0.129)***	-0.103(-0.154--0.053)****	0.059(0.005-0.113)**	0.058(0.003-0.113)**	0.01(-0.043-0.063)
Superior frontal medial area R	-0.003(-0.053-0.046)	0.066(0.016-0.116)**	-0.088(-0.138--0.038)****	0.046(-0.008-0.099)*	0.052(-0.003-0.107)*	0.011(-0.041-0.064)
Superior frontal orbital area L	0.025(-0.024-0.074)	0.072(0.022-0.123)***	-0.084(-0.134--0.033)***	0.077(0.024-0.131)***	0.054(-0.001-0.108)*	0.003(-0.049-0.055)

Superior frontal orbital area R	0.039(-0.011-0.089)	0.058(0.007-0.11)**	-0.061(-0.112--0.01)**	0.078(0.024-0.133)***	0.052(-0.003-0.108)*	-0.004(-0.057-0.049)
Fusiform gyrus L	0.028(-0.017-0.072)	0.05(0.004-0.095)**	-0.037(-0.083-0.008)	0.078(0.03-0.126)***	0.041(-0.009-0.09)	0.024(-0.024-0.071)
Fusiform gyrus R	0.045(0.003-0.088)**	0.073(0.029-0.117)***	-0.055(-0.099--0.011)**	0.09(0.043-0.137)****	0.048(0-0.096)*	0.022(-0.023-0.068)
Heschl gyrus L	0.058(0.006-0.109)**	0.107(0.055-0.16)****	-0.056(-0.109--0.003)**	0.052(-0.005-0.109)*	0.094(0.036-0.151)***	0.003(-0.053-0.058)
Heschl gyrus R	0.038(-0.014-0.09)	0.115(0.061-0.168)****	-0.053(-0.106-0)*	0.095(0.038-0.152)***	0.103(0.045-0.161)****	0.049(-0.006-0.105)*
Hippocampus L	0.044(-0.003-0.091)*	0.065(0.017-0.113)***	-0.036(-0.084-0.012)	0.085(0.034-0.136)***	0.074(0.022-0.126)***	0.052(0.002-0.102)**
Hippocampus R	0.061(0.014-0.107)**	0.068(0.02-0.117)***	-0.043(-0.091-0.005)*	0.105(0.054-0.156)****	0.082(0.03-0.134)***	0.065(0.015-0.115)**
Insula L	0.055(0.007-0.103)**	0.139(0.09-0.188)****	-0.092(-0.141--0.042)****	0.093(0.04-0.146)****	0.099(0.045-0.152)****	0.041(-0.01-0.093)
Insula R	0.06(0.012-0.109)**	0.142(0.093-0.191)****	-0.085(-0.135--0.036)****	0.11(0.058-0.163)****	0.115(0.061-0.169)****	0.05(-0.001-0.102)*
Lingual gyrus L	0.018(-0.027-0.064)	0.079(0.033-0.126)****	-0.052(-0.099--0.006)**	0.064(0.015-0.113)**	0.052(0.002-0.102)**	0.037(-0.011-0.086)
Lingual gyrus R	0.047(0.002-0.093)**	0.08(0.034-0.127)****	-0.071(-0.117--0.025)***	0.093(0.043-0.142)****	0.056(0.005-0.106)**	0.054(0.006-0.102)**
Inferior occipital lobe L	0.05(0.002-0.099)**	0.085(0.035-0.134)****	-0.045(-0.095-0.005)*	0.07(0.017-0.123)***	0.054(0-0.108)**	0(-0.052-0.051)

Inferior occipital lobe R	-0.004(-0.051-0.042)	0.06(0.012-0.107)**	-0.023(-0.07-0.025)	0.051(0-0.101)*	0.016(-0.035-0.068)	-0.016(-0.066-0.033)
Middle occipital lobe L	0.027(-0.023-0.077)	0.087(0.036-0.139)****	-0.032(-0.083-0.019)	0.051(-0.004-0.106)*	0.038(-0.018-0.094)	-0.012(-0.066-0.041)
Middle occipital lobe R	-0.008(-0.057-0.04)	0.087(0.036-0.137)****	-0.04(-0.09-0.01)	0.054(0-0.108)**	0.052(-0.003-0.106)*	0.035(-0.017-0.088)
Superior occipital lobe L	0.047(-0.004-0.097)*	0.084(0.033-0.136)***	-0.07(-0.121--0.018)***	0.078(0.023-0.133)***	0.056(0-0.112)**	0.041(-0.013-0.095)
Superior occipital lobe R	-0.005(-0.056-0.045)	0.079(0.027-0.132)***	-0.042(-0.094-0.01)	0.063(0.008-0.119)**	0.072(0.016-0.129)**	0.054(0-0.108)*
Pallidum L	0.017(-0.029-0.064)	0.059(0.01-0.107)**	-0.043(-0.091-0.005)*	0.058(0.007-0.109)**	0.03(-0.023-0.082)	0.033(-0.017-0.083)
Pallidum R	0.031(-0.015-0.078)	0.042(-0.006-0.09)*	-0.033(-0.081-0.014)	0.062(0.011-0.113)**	0.036(-0.017-0.088)	0.032(-0.018-0.082)
Paracentral lobule L	0.012(-0.039-0.062)	0.069(0.017-0.121)***	-0.061(-0.113--0.009)**	0.054(-0.001-0.109)*	0.028(-0.029-0.084)	0.035(-0.019-0.089)
Paracentral lobule R	0.042(-0.009-0.093)	0.069(0.017-0.122)***	-0.073(-0.125--0.021)***	0.08(0.024-0.136)***	0.081(0.024-0.138)***	0.025(-0.03-0.08)
Parahippocampal gyrus L	0.042(-0.002-0.086)*	0.06(0.015-0.105)***	-0.03(-0.075-0.015)	0.077(0.029-0.125)***	0.069(0.02-0.118)***	0.028(-0.019-0.075)
Parahippocampal gyrus R	0.05(0.005-0.095)**	0.068(0.022-0.114)***	-0.036(-0.082-0.01)	0.099(0.05-0.147)****	0.066(0.017-0.116)***	0.041(-0.007-0.088)*
Inferior parietal lobule L	-0.016(-0.069-0.037)	0.087(0.032-0.141)***	-0.098(-0.152--0.044)****	0.064(0.006-0.122)**	0.025(-0.034-0.085)	0.044(-0.013-0.101)

Inferior parietal lobule R	-0.035(-0.087-0.018)	0.101(0.047-0.154)****	-0.075(-0.128--0.022)***	0.065(0.008-0.122)**	0.019(-0.039-0.077)	0.003(-0.052-0.059)
Superior parietal lobule R	-0.014(-0.067-0.038)	0.104(0.05-0.158)****	-0.098(-0.152--0.044)****	0.1(0.042-0.157)****	0.05(-0.008-0.109)*	0.066(0.01-0.123)**
Superior parietal lobule L	-0.014(-0.066-0.038)	0.085(0.032-0.139)***	-0.058(-0.111--0.004)**	0.104(0.048-0.161)****	0.068(0.01-0.126)**	0.061(0.006-0.117)**
Postcentral gyrus L	-0.009(-0.06-0.042)	0.126(0.074-0.179)****	-0.1(-0.153--0.048)****	0.089(0.033-0.146)***	0.061(0.003-0.118)**	0.032(-0.023-0.087)
Postcentral gyrus R	0.032(-0.02-0.083)	0.093(0.039-0.146)****	-0.062(-0.115--0.009)**	0.085(0.028-0.142)***	0.045(-0.013-0.103)	0.039(-0.017-0.094)
Precentral gyrus L	-0.021(-0.072-0.03)	0.083(0.03-0.135)***	-0.075(-0.127--0.022)***	0.08(0.024-0.136)***	0.074(0.017-0.13)**	0.037(-0.018-0.092)
Precentral gyrus R	0.018(-0.032-0.068)	0.109(0.058-0.161)****	-0.067(-0.118--0.016)***	0.079(0.025-0.134)***	0.076(0.021-0.132)***	0.025(-0.028-0.079)
Precuneus L	0.036(-0.013-0.084)	0.088(0.038-0.138)****	-0.075(-0.125--0.025)***	0.107(0.054-0.16)****	0.063(0.009-0.117)**	0.068(0.016-0.12)**
Precuneus R	0.044(-0.005-0.092)*	0.104(0.055-0.154)****	-0.078(-0.128--0.028)***	0.109(0.056-0.162)****	0.078(0.024-0.132)***	0.07(0.019-0.122)***
Putamen L	0.025(-0.022-0.073)	0.034(-0.015-0.083)	-0.039(-0.088-0.01)	0.052(0-0.105)*	0.029(-0.025-0.082)	0.028(-0.023-0.079)
Putamen R	0.04(-0.007-0.086)*	0.051(0.003-0.099)**	-0.051(-0.099--0.003)**	0.043(-0.008-0.095)	0.041(-0.011-0.094)	0.02(-0.03-0.071)
Rectus gyrus L	0.054(0.006-0.102)**	0.094(0.044-0.143)****	-0.072(-0.122--0.022)***	0.105(0.052-0.158)****	0.072(0.018-0.126)***	0.048(-0.003-0.1)*

Rectus gyrus R	0.043(-0.005-0.092)*	0.094(0.045-0.144)****	-0.059(-0.108--0.009)**	0.114(0.061-0.166)****	0.073(0.02-0.127)***	0.059(0.008-0.111)**
Rolandic operculum L	0.022(-0.029-0.072)	0.109(0.057-0.16)****	-0.079(-0.131--0.028)***	0.064(0.009-0.119)**	0.098(0.042-0.154)****	0.011(-0.043-0.065)
Rolandic operculum R	0.058(0.008-0.108)**	0.12(0.069-0.172)****	-0.079(-0.13--0.027)***	0.092(0.037-0.148)***	0.106(0.05-0.162)****	0.039(-0.014-0.093)
Supplemental motor area L	-0.001(-0.051-0.05)	0.07(0.018-0.122)***	-0.108(-0.16--0.056)****	0.042(-0.014-0.097)	0.035(-0.021-0.092)	0.026(-0.028-0.08)
Supplemental motor area R	-0.011(-0.062-0.039)	0.08(0.028-0.132)***	-0.091(-0.143--0.039)****	0.053(-0.003-0.108)*	0.064(0.008-0.121)**	0.029(-0.025-0.084)
Supramarginal gyrus L	0.012(-0.039-0.063)	0.074(0.022-0.126)***	-0.103(-0.155--0.051)****	0.067(0.012-0.123)**	0.059(0.002-0.115)**	0.013(-0.041-0.068)
Supramarginal gyrus R	-0.01(-0.061-0.04)	0.096(0.044-0.147)****	-0.056(-0.108--0.005)**	0.067(0.013-0.122)**	0.047(-0.008-0.103)*	0.028(-0.026-0.081)
Inferior temporal gyrus L	0.008(-0.037-0.052)	0.068(0.023-0.114)***	-0.072(-0.117--0.027)***	0.061(0.012-0.109)**	0.036(-0.014-0.085)	0.009(-0.038-0.057)
Inferior temporal gyrus R	0.021(-0.022-0.065)	0.04(-0.005-0.085)*	-0.047(-0.092--0.003)**	0.08(0.033-0.128)****	0.027(-0.021-0.075)	-0.004(-0.05-0.042)
Middle temporal gyrus L	0.028(-0.019-0.076)	0.087(0.039-0.136)****	-0.055(-0.104--0.007)**	0.068(0.016-0.119)**	0.071(0.018-0.124)***	0.002(-0.049-0.052)
Middle temporal gyrus R	0.019(-0.027-0.066)	0.078(0.031-0.126)***	-0.056(-0.103--0.008)**	0.078(0.028-0.129)***	0.065(0.013-0.116)**	0.007(-0.043-0.056)

Temporal pole middle part L	-0.011(-0.06-0.038)	0.048(-0.002-0.098)*	-0.058(-0.107--0.008)**	0.023(-0.031-0.077)	0.027(-0.028-0.081)	-0.011(-0.064-0.041)
Temporal pole middle part R	0.025(-0.022-0.073)	0.036(-0.013-0.084)	-0.032(-0.08-0.017)	0.047(-0.005-0.098)*	0.041(-0.011-0.094)	-0.002(-0.052-0.048)
Temporal pole superior part L	-0.005(-0.055-0.046)	0.101(0.049-0.152)****	-0.076(-0.128--0.025)***	0.067(0.012-0.123)**	0.071(0.015-0.127)**	0.038(-0.016-0.091)
Temporal pole superior part R	0.042(-0.008-0.091)*	0.094(0.044-0.145)****	-0.051(-0.102-0)**	0.086(0.032-0.14)***	0.093(0.038-0.147)****	0.05(-0.003-0.103)*
Superior temporal gyrus L	0.049(0-0.099)**	0.104(0.053-0.154)****	-0.065(-0.115--0.015)**	0.087(0.033-0.141)***	0.103(0.048-0.158)****	0.021(-0.031-0.074)
Superior temporal gyrus R	0.035(-0.012-0.083)	0.094(0.045-0.144)****	-0.046(-0.095-0.003)*	0.08(0.028-0.133)***	0.077(0.024-0.13)***	0.037(-0.014-0.088)
Thalamus L	0.067(0.015-0.12)**	0.047(-0.007-0.101)*	-0.029(-0.083-0.024)	0.098(0.041-0.155)****	0.076(0.018-0.135)**	0.032(-0.024-0.088)
Thalamus R	0.05(-0.002-0.102)*	0.067(0.014-0.12)**	-0.017(-0.07-0.036)	0.115(0.058-0.171)****	0.072(0.014-0.13)**	0.049(-0.007-0.104)*
Cerebellum L	0.033(-0.012-0.078)	0.045(-0.001-0.091)*	-0.044(-0.091-0.002)*	0.025(-0.024-0.075)	0.025(-0.026-0.075)	0.021(-0.027-0.07)
Cerebellum R	0.04(-0.006-0.086)*	0.056(0.008-0.103)**	-0.048(-0.095--0.001)**	0.041(-0.009-0.092)	0.023(-0.028-0.075)	0.028(-0.021-0.077)

Covariates are the same as those from multiple regression analyses in the main text that do not include total GMV as a covariate.

*P < 0.1, **P < 0.05, ***P < 0.01, ****P < 0.001

Supplemental Table 2. The strength of correlations between cognitive scores and rGMV for each anatomical ROI (simple correlation coefficients in males, simple correlation coefficients in females, beta coefficients of the multiple regression analyses correction for age and sex among the entire sample).

	RAPM	digit span	Stroop interference	TBIT spatial factors	TBIT reasoning factors	TBIT perceptual factors
Total gray matter volume	0.05,0.026,0.032	0.118,0.119,0.101	-0.086,-0.106,-0.08	0.092,0.1,0.088	0.087,0.087,0.074	0.02,0.064,0.032
Amygdala L	0.059,0.051,0.046	0.107,0.092,0.086	-0.064,-0.04,-0.051	0.043,0.158,0.075	0.079,0.119,0.082	0.004,0.115,0.036
Amygdala R	0.067,0.052,0.051	0.081,0.064,0.064	-0.027,-0.083,-0.043	0.053,0.111,0.065	0.082,0.091,0.075	0.025,0.073,0.035
Angular gyrus L	0.064,-0.013,0.03	0.099,0.115,0.102	-0.056,-0.11,-0.068	0.082,0.032,0.068	0.075,0.001,0.043	0.042,0.033,0.038
Angular gyrus R	-0.002,0.025,0.007	0.075,0.141,0.094	-0.06,-0.062,-0.053	0.057,0.057,0.059	0.016,0,0.008	-0.019,0.018,-0.004
Calcarine Cortex L	0.073,0.034,0.052	0.122,0.105,0.109	-0.039,-0.073,-0.052	0.069,0.087,0.076	0.094,0.015,0.059	0.018,0.082,0.04
Calcarine Cortex R	0.074,0.076,0.069	0.125,0.083,0.103	-0.04,-0.056,-0.049	0.076,0.117,0.09	0.103,0.022,0.067	0.015,0.076,0.035
Caudate L	0.002,-0.006,-0.003	0.064,0.01,0.043	-0.021,-0.042,-0.027	0.01,0.001,0.011	0,0.042,0.014	-0.032,-0.004,-0.019
Caudate R	0.012,-0.01,0.001	0.067,0.004,0.043	-0.018,-0.03,-0.023	0.018,0.009,0.019	-0.004,0.062,0.02	-0.012,0.007,-0.004

Anterior cingulum L	0.049,0.03,0.036	0.064,0.138,0.088	-0.113,-0.065,-0.086	0.064,0.079,0.076	0.08,0.092,0.081	0.013,0.045,0.026
Anterior cingulum R	0.039,0.004,0.021	0.038,0.119,0.065	-0.117,-0.057,-0.085	0.072,0.059,0.069	0.07,0.077,0.069	0.005,0.041,0.019
Middle cingulum L	0.043,0.02,0.028	0.102,0.103,0.097	-0.121,-0.093,-0.103	0.07,0.065,0.074	0.116,0.025,0.075	0.024,0.026,0.026
Middle cingulum R	0.038,0.038,0.031	0.084,0.095,0.083	-0.122,-0.105,-0.105	0.071,0.073,0.077	0.09,0.04,0.065	0.01,0.031,0.019
Posterior cingulum L	0.019,0.048,0.026	0.081,0.077,0.074	-0.043,-0.068,-0.053	0.096,0.074,0.085	0.09,0.022,0.06	0.082,0.012,0.052
Posterior cingulum R	0.002,0.059,0.02	0.084,0.075,0.075	-0.041,-0.072,-0.049	0.07,0.069,0.069	0.051,0.006,0.03	0.027,0.005,0.019
Cuneus L	0.056,0.027,0.039	0.115,0.054,0.086	-0.088,-0.053,-0.069	0.044,0.082,0.062	0.115,-0.005,0.062	0.02,0.041,0.028
Cuneus R	0.07,-0.001,0.038	0.126,0.1,0.109	-0.071,-0.062,-0.064	0.044,0.129,0.078	0.111,0.037,0.077	0.013,0.103,0.044
Inferior frontal operculum L	0.035,-0.014,0.013	0.125,0.067,0.103	-0.101,-0.025,-0.079	0.088,0.048,0.078	0.089,0.138,0.109	0.039,0.035,0.038
Inferior frontal operculum R	0.007,0.057,0.023	0.103,0.101,0.1	-0.061,-0.031,-0.046	0.029,0.065,0.051	0.087,0.087,0.085	0.003,0.058,0.026
Inferior frontal orbital area L	0.029,0.045,0.03	0.115,0.059,0.087	-0.078,-0.041,-0.061	0.09,0.085,0.088	0.094,0.108,0.092	0.002,0.027,0.011
Inferior frontal orbital area R	0.028,0.047,0.029	0.074,0.083,0.072	-0.064,-0.074,-0.057	0.093,0.039,0.076	0.103,0.105,0.096	-0.005,0.026,0.008
Inferior frontal triangular L	0.009,-0.023,-0.006	0.092,0.069,0.081	-0.077,-0.062,-0.069	0.052,0.051,0.056	0.042,0.062,0.047	-0.003,0.028,0.009
Inferior frontal triangular R	-0.03,0.031,-0.009	0.062,0.08,0.066	-0.053,-0.079,-0.054	0.052,0.078,0.067	0.05,0.092,0.062	-0.019,0.002,-0.009

Middle frontal other areas L	0.004,-0.03,-0.011	0.083,0.124,0.093	-0.123,-0.036,-0.087	0.081,0.053,0.073	0.068,0.073,0.066	0.02,0.024,0.021
Middle frontal other areas R	0.011,0.003,0.005	0.079,0.104,0.083	-0.059,-0.064,-0.054	0.098,0.052,0.082	0.095,0.072,0.081	0.015,0.03,0.02
Middle frontal medial area L	0.058,0.027,0.04	0.069,0.103,0.077	-0.099,-0.079,-0.081	0.061,0.031,0.054	0.088,0.061,0.073	0.006,0.013,0.01
Middle frontal medial area R	0.034,0.037,0.03	0.068,0.104,0.076	-0.092,-0.075,-0.075	0.074,0.045,0.065	0.07,0.062,0.062	-0.006,0.024,0.006
Middle frontal orbital area L	0.011,0.037,0.017	0.076,0.076,0.07	-0.092,-0.061,-0.069	0.071,0.067,0.069	0.06,0.072,0.06	0.001,0.03,0.012
Middle frontal orbital area R	0.048,0.063,0.047	0.051,0.049,0.048	-0.075,-0.043,-0.047	0.067,0.02,0.054	0.051,0.028,0.039	-0.047,-0.008,-0.029
Superior frontal other areas L	-0.004,-0.061,-0.026	0.08,0.098,0.083	-0.082,-0.035,-0.064	0.049,0.03,0.047	0.071,0.098,0.078	0.026,0.023,0.025
Superior frontal other areas R	0.018,-0.025,-0.001	0.075,0.098,0.08	-0.102,-0.081,-0.09	0.057,0.052,0.058	0.077,0.078,0.074	0.02,0.036,0.025
Superior frontal medial area L	0.003,-0.022,-0.009	0.06,0.122,0.078	-0.123,-0.096,-0.103	0.06,0.048,0.059	0.075,0.042,0.058	0.002,0.02,0.01
Superior frontal medial area R	0.007,-0.014,-0.003	0.064,0.083,0.066	-0.111,-0.081,-0.088	0.042,0.045,0.046	0.047,0.071,0.052	-0.004,0.038,0.011
Superior frontal orbital area L	0.025,0.04,0.025	0.073,0.085,0.072	-0.105,-0.076,-0.084	0.075,0.075,0.077	0.071,0.038,0.054	-0.008,0.019,0.003
Superior frontal orbital area R	0.048,0.041,0.039	0.056,0.07,0.058	-0.072,-0.075,-0.061	0.084,0.058,0.078	0.075,0.025,0.052	-0.01,0.002,-0.004
Fusiform gyrus L	0.025,0.053,0.028	0.051,0.072,0.05	-0.034,-0.063,-0.037	0.089,0.084,0.078	0.055,0.04,0.041	0.025,0.032,0.024
Fusiform gyrus R	0.06,0.058,0.045	0.075,0.113,0.073	-0.043,-0.105,-0.055	0.088,0.128,0.09	0.067,0.047,0.048	0.019,0.039,0.022

Heschl gyrus L	0.082,0.03,0.058	0.126,0.082,0.107	-0.06,-0.055,-0.056	0.054,0.036,0.052	0.097,0.092,0.094	-0.014,0.03,0.003
Heschl gyrus R	0.04,0.044,0.038	0.128,0.099,0.115	-0.041,-0.065,-0.053	0.096,0.084,0.095	0.097,0.113,0.103	0.047,0.056,0.049
Hippocampus L	0.077,0.007,0.044	0.086,0.05,0.065	-0.024,-0.046,-0.036	0.098,0.09,0.085	0.078,0.091,0.074	0.044,0.092,0.052
Hippocampus R	0.083,0.045,0.061	0.069,0.096,0.068	-0.021,-0.062,-0.043	0.1,0.156,0.105	0.085,0.101,0.082	0.048,0.13,0.065
Insula L	0.071,0.055,0.055	0.16,0.136,0.139	-0.094,-0.098,-0.092	0.09,0.095,0.093	0.082,0.147,0.099	0.018,0.088,0.041
Insula R	0.068,0.075,0.06	0.141,0.175,0.142	-0.075,-0.11,-0.085	0.099,0.123,0.11	0.109,0.147,0.115	0.028,0.095,0.05
Lingual gyrus L	0.039,0.002,0.018	0.085,0.105,0.079	-0.053,-0.066,-0.052	0.066,0.074,0.064	0.08,0.033,0.052	0.013,0.094,0.037
Lingual gyrus R	0.072,0.037,0.047	0.093,0.095,0.08	-0.045,-0.129,-0.071	0.098,0.112,0.093	0.105,0.001,0.056	0.052,0.083,0.054
Inferior occipital lobe L	0.069,0.039,0.05	0.095,0.088,0.085	-0.007,-0.128,-0.045	0.072,0.074,0.07	0.048,0.076,0.054	-0.037,0.062,0
Inferior occipital lobe R	-0.005,-0.002,-0.004	0.096,0.016,0.06	-0.038,-0.009,-0.023	0.042,0.076,0.051	0.03,0.001,0.016	-0.045,0.029,-0.016
Middle occipital lobe L	0.021,0.047,0.027	0.091,0.097,0.087	-0.03,-0.052,-0.032	0.029,0.082,0.051	0.037,0.045,0.038	-0.046,0.038,-0.012
Middle occipital lobe R	-0.023,0.016,-0.008	0.097,0.088,0.087	-0.051,-0.032,-0.04	0.024,0.098,0.054	0.079,0.021,0.052	0.039,0.034,0.035
Superior occipital lobe L	0.027,0.091,0.047	0.101,0.068,0.084	-0.08,-0.055,-0.07	0.055,0.11,0.078	0.079,0.029,0.056	0.019,0.082,0.041
Superior occipital lobe R	-0.005,-0.003,-0.005	0.09,0.07,0.079	-0.06,-0.01,-0.042	0.026,0.12,0.063	0.078,0.069,0.072	0.027,0.106,0.054

Pallidum L	0.018,0.031,0.017	0.085,0.031,0.059	-0.034,-0.079,-0.043	0.027,0.114,0.058	0.026,0.048,0.03	-0.011,0.123,0.033
Pallidum R	0.02,0.073,0.031	0.069,0.006,0.042	-0.016,-0.077,-0.033	0.026,0.123,0.062	0.037,0.048,0.036	-0.011,0.117,0.032
Paracentral lobule L	0.033,-0.014,0.012	0.078,0.062,0.069	-0.064,-0.059,-0.061	0.069,0.02,0.054	0.047,0.004,0.028	0.021,0.061,0.035
Paracentral lobule R	0.048,0.042,0.042	0.082,0.054,0.069	-0.097,-0.026,-0.073	0.069,0.093,0.08	0.089,0.072,0.081	-0.004,0.076,0.025
Parahippocampal gyrus L	0.057,0.046,0.042	0.069,0.078,0.06	-0.037,-0.025,-0.03	0.073,0.113,0.077	0.074,0.093,0.069	0,0.091,0.028
Parahippocampal gyrus R	0.07,0.046,0.05	0.068,0.104,0.068	-0.015,-0.062,-0.036	0.115,0.116,0.099	0.078,0.078,0.066	0.039,0.066,0.041
Inferior parietal lobule L	0.009,-0.046,-0.016	0.08,0.095,0.087	-0.118,-0.066,-0.098	0.096,-0.004,0.064	0.017,0.04,0.025	0.055,0.024,0.044
Inferior parietal lobule R	-0.021,-0.053,-0.035	0.105,0.099,0.101	-0.05,-0.117,-0.075	0.077,0.037,0.065	-0.004,0.053,0.019	-0.015,0.029,0.003
Superior parietal lobule R	0,-0.033,-0.014	0.113,0.091,0.104	-0.087,-0.098,-0.098	0.115,0.064,0.1	0.074,0.015,0.05	0.075,0.052,0.066
Superior parietal lobule L	0.011,-0.052,-0.014	0.097,0.07,0.085	-0.049,-0.057,-0.058	0.122,0.073,0.104	0.096,0.027,0.068	0.054,0.078,0.061
Postcentral gyrus L	-0.009,-0.003,-0.009	0.129,0.131,0.126	-0.119,-0.08,-0.1	0.065,0.117,0.089	0.072,0.048,0.061	0.01,0.067,0.032
Postcentral gyrus R	0.04,0.026,0.032	0.091,0.101,0.093	-0.068,-0.051,-0.062	0.1,0.05,0.085	0.057,0.03,0.045	0.03,0.054,0.039
Precentral gyrus L	0.012,-0.071,-0.021	0.102,0.059,0.083	-0.089,-0.044,-0.075	0.111,0.03,0.08	0.076,0.074,0.074	0.067,-0.011,0.037
Precentral gyrus R	0.015,0.031,0.018	0.126,0.097,0.109	-0.077,-0.039,-0.067	0.084,0.073,0.079	0.062,0.108,0.076	0.001,0.071,0.025

Precuneus L	0.042,0.043,0.036	0.105,0.078,0.088	-0.065,-0.095,-0.075	0.12,0.087,0.107	0.098,0.024,0.063	0.085,0.052,0.068
Precuneus R	0.038,0.072,0.044	0.103,0.131,0.104	-0.063,-0.106,-0.078	0.098,0.129,0.109	0.098,0.065,0.078	0.074,0.079,0.07
Putamen L	0.005,0.075,0.025	0.064,-0.009,0.034	-0.013,-0.103,-0.039	0.02,0.091,0.052	0.026,0.044,0.029	-0.026,0.121,0.028
Putamen R	0.021,0.096,0.04	0.073,0.026,0.051	-0.028,-0.122,-0.051	-0.002,0.102,0.043	0.041,0.059,0.041	-0.036,0.119,0.02
Rectus gyrus L	0.058,0.071,0.054	0.089,0.122,0.094	-0.061,-0.095,-0.072	0.118,0.085,0.105	0.086,0.066,0.072	0.056,0.043,0.048
Rectus gyrus R	0.055,0.045,0.043	0.097,0.112,0.094	-0.038,-0.094,-0.059	0.137,0.081,0.114	0.112,0.032,0.073	0.076,0.045,0.059
Rolandic operculum L	0.046,-0.008,0.022	0.126,0.091,0.109	-0.077,-0.09,-0.079	0.066,0.049,0.064	0.105,0.098,0.098	0.01,0.01,0.011
Rolandic operculum R	0.058,0.076,0.058	0.119,0.137,0.12	-0.064,-0.101,-0.079	0.077,0.109,0.092	0.108,0.113,0.106	0.025,0.067,0.039
Supplemental motor area L	0.007,-0.007,-0.001	0.059,0.097,0.07	-0.122,-0.087,-0.108	0.051,0.015,0.042	0.035,0.042,0.035	0.001,0.07,0.026
Supplemental motor area R	0.016,-0.054,-0.011	0.085,0.083,0.08	-0.109,-0.067,-0.091	0.055,0.042,0.053	0.051,0.093,0.064	0.02,0.049,0.029
Supramarginal gyrus L	0.035,-0.016,0.012	0.084,0.064,0.074	-0.126,-0.085,-0.103	0.094,0.008,0.067	0.04,0.097,0.059	0.028,-0.016,0.013
Supramarginal gyrus R	-0.014,-0.001,-0.01	0.095,0.111,0.096	-0.049,-0.083,-0.056	0.042,0.105,0.067	0.016,0.107,0.047	0.002,0.076,0.028
Inferior temporal gyrus L	0.005,0.023,0.008	0.081,0.081,0.068	-0.063,-0.127,-0.072	0.065,0.07,0.061	0.023,0.074,0.036	0.019,-0.006,0.009
Inferior temporal gyrus R	0.019,0.043,0.021	0.036,0.071,0.04	-0.033,-0.103,-0.047	0.088,0.105,0.08	0.021,0.052,0.027	-0.031,0.039,-0.004

Middle temporal gyrus L	0.04,0.027,0.028	0.092,0.104,0.087	-0.053,-0.075,-0.055	0.051,0.094,0.068	0.091,0.06,0.071	-0.001,0.003,0.002
Middle temporal gyrus R	0.017,0.035,0.019	0.07,0.125,0.078	-0.055,-0.075,-0.056	0.075,0.105,0.078	0.075,0.071,0.065	-0.018,0.052,0.007
Temporal pole middle part L	0.019,-0.054,-0.011	0.046,0.061,0.048	-0.08,-0.063,-0.058	0.014,0.022,0.023	0.045,0.007,0.027	0.003,-0.045,-0.011
Temporal pole middle part R	0.028,0.035,0.025	0.049,0.025,0.036	-0.025,-0.078,-0.032	0.037,0.065,0.047	0.066,0.018,0.041	-0.003,-0.004,-0.002
Temporal pole superior part L	0.009,-0.016,-0.005	0.122,0.075,0.101	-0.079,-0.076,-0.076	0.061,0.057,0.067	0.083,0.063,0.071	0.044,0.026,0.038
Temporal pole superior part R	0.025,0.082,0.042	0.103,0.097,0.094	-0.031,-0.1,-0.051	0.078,0.098,0.086	0.093,0.107,0.093	0.04,0.075,0.05
Superior temporal gyrus L	0.059,0.053,0.049	0.132,0.072,0.104	-0.096,-0.027,-0.065	0.095,0.068,0.087	0.117,0.098,0.103	0.034,-0.001,0.021
Superior temporal gyrus R	0.037,0.051,0.035	0.112,0.088,0.094	-0.059,-0.034,-0.046	0.09,0.071,0.08	0.079,0.093,0.077	0.033,0.053,0.037
Thalamus L	0.089,0.043,0.067	0.064,0.022,0.047	0.003,-0.055,-0.029	0.109,0.081,0.098	0.1,0.043,0.076	0.038,0.025,0.032
Thalamus R	0.063,0.038,0.05	0.087,0.043,0.067	0.003,-0.025,-0.017	0.137,0.082,0.115	0.085,0.056,0.072	0.076,0.012,0.049
Cerebellum L	0.068,0,0.033	0.05,0.056,0.045	-0.023,-0.112,-0.044	0.012,0.037,0.025	0.013,0.06,0.025	0.007,0.054,0.021
Cerebellum R	0.077,0.004,0.04	0.065,0.061,0.056	-0.03,-0.1,-0.048	0.037,0.045,0.041	0.011,0.057,0.023	0.012,0.067,0.028

Supplemental Table 3. Brain regions exhibiting significant correlations between general intelligence factor that is calculated from 5 tasks and rGMV

No	Included gray matter areas*(number of significant voxels in left and right side of each anatomical area)	x	y	z	TFCE value	Corrected *p*-value (TFCE, FWE)	Cluster size (voxel)
1	Amygdala (L:522, R:592)/Angular gyrus (L:1192, R:15)/Calcarine Cortex (L:3205, R:3085)/Caudate (L:300, R:447)/Anterior cingulum (L:2596, R:2075)/Middle cingulum (L:3114, R:2939)/Posterior cingulum (L:925, R:649)/Cuneus (L:1733, R:1787)/Inferior frontal operculum (L:1529, R:1417)/Inferior frontal orbital area (L:3584, R:2582)/Inferior frontal triangular (L:1235, R:894)/Middle frontal medial area (L:1342, R:1358)/Middle frontal orbital area (L:1322, R:435)/Middle frontal other areas (L:1746, R:3357)/Superior frontal medial area (L:1230, R:941)/Superior frontal orbital area (L:1236, R:928)/Superior frontal other areas (L:1349, R:1477)/Fusiform gyrus (L:2196, R:3627)/Heschl gyrus (L:516, R:586)/Hippocampus (L:2041, R:2180)/Insula (L:4536, R:4408)/Lingual gyrus (L:2465, R:4279)/Inferior occipital	43.5	3	16.5	5719.49	0.0006	209913

	lobe (L:804, R:296)/Middle occipital lobe (L:1273, R:1800)/Superior occipital lobe (L:1519, R:1746)/Pallidum (L:177, R:325)/Paracentral lobule (L:704, R:1222)/Parahippocampal gyrus (L:1725, R:2198)/Inferior parietal lobule (L:1260, R:59)/Superior parietal lobule (L:1991, R:1807)/Postcentral gyrus (L:2948, R:3246)/Precentral gyrus (L:2460, R:2998)/Precuneus (L:5585, R:5745)/Putamen (L:1395, R:1600)/Rectus gyrus (L:2110, R:1857)/Rolandic operculum (L:1862, R:2652)/Supplemental motor area (L:823, R:801)/Supramarginal gyrus (L:1236, R:614)/Inferior temporal gyrus (L:743, R:1528)/Middle temporal gyrus (L:3603, R:3487)/Temporal pole (L:1777, R:2669)/Superior temporal gyrus (L:4512, R:4464)/Thalamus (L:2047, R:2092)/Cerebellum (L:1017, R:2706)/						
2	Inferior temporal gyrus (R:2)/	57	-42	-19.5	1452.62	0.042	2
3	None	-10.5	25.5	9	1452.62	0.042	1
4	Inferior temporal gyrus (L:95)/	-46.5	-9	-37.5	1361.66	0.0496	110
5	Middle cingulum (R:12)/Supplemental motor area (L:23, R:136)/	7.5	18	52.5	1361.53	0.0496	156
6	None	34.5	66	-10.5	1353.7	0.050	3
7	None	73.5	-39	15	1353.69	0.050	1

*Labeling of anatomical gray matter regions was based on the WFU PickAtlas Tool (http://www.fmri.wfubmc.edu/cms/software#PickAtlas/) [10, 11] and the PickAtlas automated anatomical labeling atlas option [12]. Temporal pole and cerebellum include all subregions of these areas in the atlas.

Supplemental Table 4. Brain regions exhibiting significant correlations between digit span performance that is independent of general intelligence factor that is calculated from other tasks.

No	Included gray matter areas*(number of significant voxels in left and right side of each anatomical area)	x	y	z	TFCE value	Corrected *p*-value (TFCE, FWE)	Cluster size (voxel)
1	Amygdala (L:483)/Angular gyrus (L:560)/Calcarine Cortex (L:82)/Caudate (L:91)/Inferior frontal operculum (L:1075)/Inferior frontal orbital area (L:1579)/Inferior frontal triangular (L:1107)/Middle frontal orbital area (L:3)/Middle frontal other areas (L:1)/Superior frontal orbital area (L:99)/Fusiform gyrus (L:84)/Heschl gyrus (L:544)/Hippocampus (L:380)/Insula (L:4368)/Lingual gyrus (L:486)/Inferior occipital lobe (L:325)/Middle occipital lobe (L:1773)/Pallidum (L:97)/Parahippocampal gyrus (L:603)/Inferior parietal lobule (L:112)/Postcentral gyrus (L:3026)/Precentral gyrus (L:1473)/Putamen (L:627)/Rectus gyrus (L:56)/Rolandic operculum (L:1960)/Supramarginal gyrus (L:288)/Inferior temporal gyrus	-40.5	12	-1.5	3508.28	0.0016	37022

	(L:631)/Middle temporal gyrus (L:2861)/Temporal pole (L:1940)/Superior temporal gyrus (L:3123)/Cerebellum (L:225)/						
2	Amygdala (R:194)/Angular gyrus (R:2042)/Calcarine Cortex (L:2354, R:1071)/Caudate (R:4)/Anterior cingulum (L:569, R:176)/Middle cingulum (L:1792, R:1151)/Posterior cingulum (L:268, R:347)/Cuneus (L:932, R:1118)/Inferior frontal operculum (R:1571)/Inferior frontal orbital area (L:1, R:200)/Inferior frontal triangular (R:746)/Middle frontal medial area (L:406, R:225)/Middle frontal orbital area (L:67)/Middle frontal other areas (R:186)/Superior frontal medial area (L:760, R:279)/Superior frontal orbital area (L:197, R:256)/Superior frontal other areas (R:198)/Fusiform gyrus (R:223)/Heschl gyrus (R:586)/Hippocampus (L:1, R:239)/Insula (R:4158)/Lingual gyrus (L:958, R:733)/Inferior occipital lobe (R:119)/Middle occipital lobe (L:80, R:2182)/Superior occipital lobe (L:357, R:980)/Paracentral lobule (L:482, R:77)/Parahippocampal gyrus (L:19, R:423)/Inferior parietal lobule (L:5, R:2088)/Superior parietal lobule (L:204, R:754)/Postcentral gyrus (L:821, R:1038)/Precentral gyrus (L:2, R:2521)/Precuneus (L:1009, R:1055)/Putamen (R:1003)/Rectus gyrus (L:851, R:901)/Rolandic operculum (R:2042)/Supplemental motor area (L:380, R:511)/Supramarginal gyrus	45	6	-1.5	3178.84	0.003	62557

	(R:1648)/Inferior temporal gyrus (R:1092)/Middle temporal gyrus (R:4207)/Temporal pole (R:785)/Superior temporal gyrus (R:3239)/Thalamus (R:85)/Cerebellum (L:88, R:87)/						
3	Fusiform gyrus (R:1056)/Lingual gyrus (R:95)/Inferior occipital lobe (R:551)/Cerebellum (R:2946)/	34.5	-67.5	-12	1879.72	0.0198	4967
4	Inferior frontal orbital area (L:4)/Inferior frontal triangular (L:219)/Middle frontal orbital area (L:72)/Middle frontal other areas (L:887)/Superior frontal other areas (L:83)/	-45	48	4.5	1563.15	0.0352	1290
5	Cuneus (L:14)/Superior occipital lobe (L:37)/Superior parietal lobule (L:11)/Precuneus (L:163)/	-16.5	-64.5	37.5	1421.34	0.0470	225
6	Cerebellum (R:87)/	24	-39	-55.5	1412.08	0.0476	122
7	Inferior frontal operculum (R:11)/Middle frontal other areas (R:22)/Precentral gyrus (R:3)/	31.5	6	36	1404.24	0.0488	60
8	Inferior parietal lobule (L:20)/	-27	-55.5	40.5	1396.67	0.049399994	20
9	Cerebellum (R:44)/	36	-60	-63	1395.54	0.049599998	47
10	Inferior parietal lobule (L:4)/	-43.5	-54	58.5	1392.82	0.049999995	4

Supplemental Table 5. Brain regions exhibiting significant correlations between general intelligence factor that is calculated from four tasks other than the digit span performance.

No	Included gray matter areas*(number of significant voxels in left and right side of each anatomical area)	x	y	z	TFCE value	Corrected *p*-value (TFCE, FWE)	Cluster size (voxel)
1	Amygdala (L:505, R:575)/Angular gyrus (L:831)/Calcarine Cortex (L:2389, R:2629)/Caudate (L:240, R:386)/Anterior cingulum (L:2217, R:1528)/Middle cingulum (L:2287, R:1799)/Posterior cingulum (L:747, R:339)/Cuneus (L:1433, R:1569)/Inferior frontal operculum (L:1181, R:914)/Inferior frontal orbital area (L:3032, R:2248)/Inferior frontal triangular (L:905, R:652)/Middle frontal medial area (L:1075, R:960)/Middle frontal orbital area (L:914, R:253)/Middle frontal other areas (L:869)/Superior frontal medial area (L:762, R:605)/Superior frontal orbital area (L:915, R:790)/Superior frontal other areas (L:632, R:603)/Fusiform gyrus (L:1824, R:3118)/Heschl gyrus (L:269, R:582)/Hippocampus (L:1929, R:2142)/Insula (L:4248, R:4243)/Lingual gyrus (L:1864, R:3697)/Inferior occipital lobe (L:183, R:123)/Middle occipital	3	-70.5	48	4631.91	0.0008	164379

	lobe (L:380, R:1223)/Superior occipital lobe (L:984, R:1388)/Pallidum (L:65, R:275)/Paracentral lobule (L:426, R:1024)/Parahippocampal gyrus (L:1471, R:2099)/Inferior parietal lobule (L:1040, R:20)/Superior parietal lobule (L:1619, R:1532)/Postcentral gyrus (L:1407, R:2702)/Precentral gyrus (L:1625, R:1366)/Precuneus (L:5141, R:5402)/Putamen (L:1091, R:1289)/Rectus gyrus (L:1996, R:1778)/Rolandic operculum (L:1258, R:2440)/Supplemental motor area (L:467, R:342)/Supramarginal gyrus (L:1070, R:178)/Inferior temporal gyrus (L:225, R:1118)/Middle temporal gyrus (L:2690, R:2825)/Temporal pole (L:1355, R:2459)/Superior temporal gyrus (L:3385, R:4048)/Thalamus (L:2009, R:2094)/Cerebellum (L:260, R:2530)/						
2	Calcarine Cortex (L:133)/Lingual gyrus (L:28)/Inferior occipital lobe (L:300)/Middle occipital lobe (L:387)/Superior occipital lobe (L:138)/	-21	-103.5	-7.5	1640	0.0322	1407
3	Middle frontal other areas (R:824)/Superior frontal other areas (R:44)/	34.5	49.5	16.5	1422.49	0.0460	889
4	Middle frontal other areas (R:6)/Superior frontal other areas (R:3)/	31.5	61.5	21	1376.21	0.0494	11

Supplemental Table 6. Beta coefficients (95% confidence intervals) from multiple regression analyses of associations between cognitive scores and rGMV for each anatomical ROI.

	g factor calculated from five tasks	digit span independent of g factor	g factor calculated from four tasks other than digit span task
Total gray matter volume	0.087(0.038-0.136)****	0.07(0.022-0.117)***	0.077(0.029-0.126)***
Amygdala L	0.09(0.041-0.139)****	0.072(0.024-0.12)***	0.079(0.03-0.129)***
Amygdala R	0.078(0.028-0.127)***	0.044(-0.004-0.093)*	0.071(0.022-0.121)***
Angular gyrus L	0.073(0.017-0.13)**	0.061(0.006-0.116)**	0.065(0.009-0.121)**
Angular gyrus R	0.043(-0.012-0.098)	0.086(0.032-0.14)***	0.031(-0.024-0.086)
Calcarine Cortex L	0.089(0.034-0.143)***	0.073(0.02-0.127)***	0.078(0.024-0.133)***
Calcarine Cortex R	0.1(0.045-0.154)****	0.063(0.01-0.117)**	0.091(0.036-0.145)***
Caudate L	0.006(-0.049-0.062)	0.034(-0.02-0.088)	0.001(-0.055-0.056)
Caudate R	0.016(-0.04-0.072)	0.027(-0.028-0.081)	0.012(-0.044-0.067)
Anterior cingulum L	0.083(0.027-0.138)***	0.054(0-0.108)**	0.075(0.02-0.13)***

Anterior cingulum R	0.066(0.011-0.12)**	0.034(-0.019-0.087)	0.061(0.007-0.116)**
Middle cingulum L	0.077(0.023-0.131)***	0.065(0.012-0.118)**	0.068(0.014-0.122)**
Middle cingulum R	0.071(0.018-0.125)***	0.048(-0.005-0.1)*	0.065(0.011-0.119)**
Posterior cingulum L	0.087(0.033-0.141)***	0.047(-0.005-0.1)*	0.081(0.027-0.135)***
Posterior cingulum R	0.062(0.008-0.116)**	0.062(0.009-0.114)**	0.054(0-0.107)*
Cuneus L	0.073(0.018-0.127)***	0.059(0.006-0.113)**	0.064(0.009-0.118)**
Cuneus R	0.091(0.037-0.146)***	0.077(0.024-0.13)***	0.08(0.026-0.135)***
Inferior frontal operculum L	0.093(0.035-0.15)***	0.074(0.018-0.13)***	0.082(0.025-0.139)***
Inferior frontal operculum R	0.071(0.014-0.127)**	0.07(0.015-0.125)**	0.06(0.004-0.116)**
Inferior frontal orbital area L	0.087(0.034-0.14)***	0.066(0.014-0.118)**	0.078(0.025-0.131)***
Inferior frontal orbital area R	0.078(0.025-0.131)***	0.048(-0.004-0.1)*	0.071(0.018-0.125)***
Inferior frontal triangular L	0.049(-0.006-0.105)*	0.062(0.007-0.116)**	0.041(-0.015-0.096)
Inferior frontal triangular R	0.041(-0.013-0.096)	0.049(-0.005-0.102)*	0.035(-0.019-0.09)
Middle frontal other areas L	0.062(0.007-0.117)**	0.056(0.002-0.11)**	0.055(0-0.11)*
Middle frontal other areas R	0.07(0.016-0.124)**	0.041(-0.012-0.095)	0.065(0.011-0.119)**
Middle frontal medial area L	0.069(0.014-0.123)**	0.054(0.001-0.107)**	0.061(0.006-0.115)**

Middle frontal medial area R	0.063(0.01-0.117)**	0.048(-0.005-0.101)*	0.057(0.003-0.11)**
Middle frontal orbital area L	0.065(0.012-0.119)**	0.047(-0.006-0.099)*	0.059(0.006-0.112)**
Middle frontal orbital area R	0.039(-0.015-0.094)	0.026(-0.028-0.079)	0.035(-0.019-0.09)
Superior frontal other areas L	0.052(-0.004-0.107)*	0.049(-0.005-0.103)*	0.045(-0.011-0.1)
Superior frontal other areas R	0.059(0.004-0.114)**	0.044(-0.01-0.097)	0.053(-0.002-0.108)*
Superior frontal medial area L	0.05(-0.005-0.104)*	0.05(-0.003-0.103)*	0.043(-0.011-0.097)
Superior frontal medial area R	0.044(-0.01-0.098)	0.037(-0.015-0.09)	0.039(-0.015-0.093)
Superior frontal orbital area L	0.063(0.009-0.116)**	0.045(-0.007-0.097)*	0.057(0.003-0.11)**
Superior frontal orbital area R	0.062(0.008-0.117)**	0.035(-0.018-0.089)	0.058(0.003-0.112)**
Fusiform gyrus L	0.064(0.016-0.112)***	0.036(-0.011-0.084)	0.06(0.012-0.108)**
Fusiform gyrus R	0.079(0.032-0.126)***	0.061(0.015-0.107)***	0.071(0.024-0.118)***
Heschl gyrus L	0.075(0.018-0.131)***	0.089(0.034-0.144)***	0.061(0.004-0.117)**
Heschl gyrus R	0.109(0.052-0.166)****	0.093(0.038-0.149)***	0.096(0.039-0.152)****
Hippocampus L	0.091(0.04-0.142)****	0.047(-0.003-0.096)*	0.085(0.034-0.136)***
Hippocampus R	0.108(0.057-0.159)****	0.04(-0.01-0.09)	0.104(0.053-0.155)****
Insula L	0.114(0.061-0.166)****	0.115(0.064-0.166)****	0.097(0.045-0.149)****

Insula R	0.129(0.077-0.182)****	0.11(0.059-0.161)****	0.113(0.061-0.166)****
Lingual gyrus L	0.068(0.018-0.117)***	0.055(0.007-0.103)**	0.06(0.011-0.109)**
Lingual gyrus R	0.093(0.043-0.142)****	0.048(0-0.097)*	0.087(0.038-0.136)****
Inferior occipital lobe L	0.069(0.016-0.121)**	0.055(0.003-0.107)**	0.061(0.008-0.113)**
Inferior occipital lobe R	0.034(-0.017-0.085)	0.06(0.011-0.11)**	0.025(-0.026-0.076)
Middle occipital lobe L	0.044(-0.011-0.099)	0.061(0.007-0.114)**	0.035(-0.02-0.089)
Middle occipital lobe R	0.063(0.01-0.117)**	0.07(0.018-0.123)***	0.053(0-0.107)*
Superior occipital lobe L	0.083(0.028-0.138)***	0.046(-0.008-0.1)*	0.077(0.022-0.132)***
Superior occipital lobe R	0.083(0.028-0.139)***	0.056(0.002-0.11)**	0.076(0.02-0.131)***
Pallidum L	0.054(0.003-0.106)**	0.051(0.001-0.101)**	0.047(-0.004-0.099)*
Pallidum R	0.057(0.005-0.108)**	0.025(-0.026-0.075)	0.054(0.003-0.105)**
Paracentral lobule L	0.052(-0.003-0.108)*	0.049(-0.006-0.103)*	0.046(-0.01-0.101)
Paracentral lobule R	0.082(0.026-0.139)***	0.032(-0.022-0.087)	0.078(0.022-0.134)***
Parahippocampal gyrus L	0.077(0.029-0.125)***	0.044(-0.003-0.091)*	0.071(0.023-0.119)***
Parahippocampal gyrus R	0.091(0.043-0.14)****	0.045(-0.003-0.092)*	0.086(0.038-0.135)****
Inferior parietal lobule L	0.052(-0.006-0.11)*	0.046(-0.011-0.103)	0.047(-0.011-0.105)

Inferior parietal lobule R	0.037(-0.02-0.094)	0.095(0.039-0.15)****	0.024(-0.033-0.08)
Superior parietal lobule R	0.086(0.028-0.144)***	0.057(0.001-0.114)**	0.08(0.022-0.137)***
Superior parietal lobule L	0.092(0.035-0.149)***	0.055(-0.001-0.111)*	0.086(0.029-0.143)***
Postcentral gyrus L	0.08(0.024-0.136)***	0.099(0.044-0.153)****	0.066(0.01-0.122)**
Postcentral gyrus R	0.076(0.019-0.133)***	0.046(-0.01-0.101)	0.07(0.014-0.127)**
Precentral gyrus L	0.066(0.01-0.122)**	0.044(-0.011-0.098)	0.061(0.005-0.117)**
Precentral gyrus R	0.08(0.025-0.135)***	0.077(0.023-0.13)***	0.069(0.015-0.124)**
Precuneus L	0.106(0.053-0.159)****	0.048(-0.004-0.1)*	0.101(0.048-0.154)****
Precuneus R	0.115(0.062-0.168)****	0.062(0.01-0.113)**	0.107(0.055-0.16)****
Putamen L	0.048(-0.005-0.1)*	0.025(-0.027-0.076)	0.045(-0.008-0.097)*
Putamen R	0.051(-0.001-0.102)*	0.039(-0.011-0.09)	0.045(-0.007-0.096)*
Rectus gyrus L	0.104(0.051-0.156)****	0.063(0.012-0.115)**	0.095(0.043-0.148)****
Rectus gyrus R	0.111(0.058-0.163)****	0.063(0.012-0.115)**	0.103(0.051-0.155)****
Rolandic operculum L	0.077(0.022-0.133)***	0.096(0.042-0.149)****	0.063(0.008-0.118)**
Rolandic operculum R	0.11(0.055-0.165)****	0.088(0.034-0.142)***	0.097(0.042-0.152)****
Supplemental motor area L	0.038(-0.018-0.094)	0.047(-0.008-0.101)*	0.032(-0.024-0.087)

Supplemental motor area R	0.053(-0.003-0.108)*	0.057(0.003-0.112)**	0.045(-0.01-0.1)
Supramarginal gyrus L	0.056(0.001-0.112)**	0.044(-0.01-0.099)	0.051(-0.005-0.106)*
Supramarginal gyrus R	0.053(-0.001-0.108)*	0.072(0.019-0.126)***	0.044(-0.011-0.098)
Inferior temporal gyrus L	0.047(-0.001-0.096)*	0.057(0.009-0.104)**	0.039(-0.009-0.088)
Inferior temporal gyrus R	0.046(-0.002-0.093)*	0.034(-0.012-0.081)	0.042(-0.005-0.089)*
Middle temporal gyrus L	0.066(0.014-0.118)**	0.057(0.007-0.108)**	0.058(0.006-0.11)**
Middle temporal gyrus R	0.067(0.017-0.118)***	0.066(0.017-0.116)***	0.058(0.008-0.109)**
Temporal pole middle part L	0.021(-0.032-0.075)	0.049(-0.003-0.101)*	0.013(-0.04-0.067)
Temporal pole middle part R	0.037(-0.014-0.089)	0.024(-0.027-0.074)	0.034(-0.017-0.086)
Temporal pole superior part L	0.077(0.021-0.132)***	0.078(0.024-0.132)***	0.066(0.011-0.12)**
Temporal pole superior part R	0.096(0.042-0.15)****	0.055(0.003-0.108)**	0.089(0.035-0.142)***
Superior temporal gyrus L	0.099(0.045-0.153)****	0.076(0.023-0.128)***	0.088(0.034-0.141)***
Superior temporal gyrus R	0.087(0.035-0.14)***	0.068(0.016-0.119)***	0.078(0.026-0.13)***
Thalamus L	0.092(0.035-0.15)***	0.019(-0.037-0.075)	0.091(0.033-0.148)***
Thalamus R	0.105(0.049-0.162)****	0.035(-0.021-0.09)	0.102(0.045-0.159)****
Cerebellum L	0.037(-0.013-0.086)	0.034(-0.014-0.083)	0.031(-0.018-0.081)

Cerebellum R	0.049(-0.001-0.099)*	0.051(0.002-0.1)**	0.042(-0.009-0.092)

Covariates are the same as those from multiple regression analyses in the main text that do not include total GMV as a covariate.

*P < 0.1, **P < 0.05, ***P < 0.01, ****P < 0.001

Supplemental Table 7. The strength of correlations between cognitive scores and rGMV for each anatomical ROI (simple correlation coefficients in males, simple correlation coefficients in females, beta coefficients of the multiple regression analyses correction for age and sex among the entire sample).

	g factor calculated from five tasks	digit span independent of g factor	g factor calculated from four tasks other than digit span task
Total gray matter volume	0.123,0.118,0.087	0.054,0.011,0.07	0.116,0.118,0.077
Amygdala L	0.072,0.163,0.09	0.099,0.057,0.072	0.058,0.155,0.079
Amygdala R	0.078,0.112,0.078	0.057,0.042,0.044	0.071,0.107,0.071
Angular gyrus L	0.094,0.041,0.073	0.038,0.105,0.061	0.091,0.027,0.065
Angular gyrus R	0.032,0.064,0.043	0.069,0.132,0.086	0.025,0.04,0.031
Calcarine Cortex L	0.092,0.093,0.089	0.08,0.075,0.073	0.082,0.083,0.078
Calcarine Cortex R	0.097,0.116,0.1	0.079,0.048,0.063	0.087,0.111,0.091
Caudate L	0.003,0.008,0.006	0.05,0.014,0.034	-0.002,0.012,0.001
Caudate R	0.015,0.015,0.016	0.045,-0.001,0.027	0.01,0.021,0.012

Anterior cingulum L	0.069,0.105,0.083	0.025,0.112,0.054	0.07,0.091,0.075
Anterior cingulum R	0.059,0.081,0.066	-0.007,0.109,0.034	0.065,0.069,0.061
Middle cingulum L	0.09,0.057,0.077	0.06,0.086,0.065	0.086,0.051,0.068
Middle cingulum R	0.074,0.071,0.071	0.04,0.072,0.048	0.073,0.067,0.065
Posterior cingulum L	0.108,0.066,0.087	0.044,0.064,0.047	0.106,0.057,0.081
Posterior cingulum R	0.062,0.07,0.062	0.058,0.085,0.062	0.059,0.057,0.054
Cuneus L	0.084,0.059,0.073	0.076,0.042,0.059	0.074,0.054,0.064
Cuneus R	0.087,0.107,0.091	0.086,0.076,0.077	0.071,0.099,0.08
Inferior frontal operculum L	0.097,0.082,0.093	0.089,0.054,0.074	0.09,0.073,0.082
Inferior frontal operculum R	0.053,0.094,0.071	0.071,0.076,0.07	0.042,0.086,0.06
Inferior frontal orbital area L	0.087,0.103,0.087	0.095,0.03,0.066	0.076,0.1,0.078
Inferior frontal orbital area R	0.08,0.083,0.078	0.049,0.059,0.048	0.075,0.073,0.071
Inferior frontal triangular L	0.044,0.058,0.049	0.066,0.064,0.062	0.038,0.049,0.041
Inferior frontal triangular R	0.021,0.075,0.041	0.046,0.065,0.049	0.018,0.066,0.035
Middle frontal other areas L	0.062,0.065,0.062	0.033,0.106,0.056	0.062,0.049,0.055
Middle frontal other areas R	0.075,0.068,0.07	0.021,0.087,0.041	0.077,0.056,0.065

Middle frontal medial area L	0.076,0.062,0.069	0.038,0.094,0.054	0.071,0.047,0.061
Middle frontal medial area R	0.061,0.074,0.063	0.031,0.09,0.048	0.06,0.06,0.057
Middle frontal orbital area L	0.061,0.083,0.065	0.054,0.045,0.047	0.055,0.075,0.059
Middle frontal orbital area R	0.04,0.038,0.039	0.026,0.031,0.026	0.04,0.03,0.035
Superior frontal other areas L	0.05,0.055,0.052	0.034,0.086,0.049	0.049,0.04,0.045
Superior frontal other areas R	0.057,0.067,0.059	0.027,0.082,0.044	0.058,0.052,0.053
Superior frontal medial area L	0.049,0.055,0.05	0.019,0.117,0.05	0.051,0.038,0.043
Superior frontal medial area R	0.033,0.067,0.044	0.027,0.066,0.037	0.033,0.055,0.039
Superior frontal orbital area L	0.058,0.077,0.063	0.043,0.061,0.045	0.055,0.068,0.057
Superior frontal orbital area R	0.069,0.053,0.062	0.028,0.056,0.035	0.069,0.045,0.058
Fusiform gyrus L	0.072,0.079,0.064	0.035,0.06,0.036	0.07,0.072,0.06
Fusiform gyrus R	0.087,0.107,0.079	0.069,0.087,0.061	0.08,0.093,0.071
Heschl gyrus L	0.081,0.064,0.075	0.097,0.083,0.089	0.065,0.052,0.061
Heschl gyrus R	0.111,0.105,0.109	0.11,0.07,0.093	0.096,0.096,0.096
Hippocampus L	0.108,0.095,0.091	0.068,0.022,0.047	0.099,0.096,0.085
Hippocampus R	0.108,0.152,0.108	0.038,0.057,0.04	0.105,0.15,0.104

Insula L	0.106,0.145,0.114	0.136,0.113,0.115	0.088,0.132,0.097
Insula R	0.119,0.163,0.129	0.11,0.139,0.11	0.105,0.148,0.113
Lingual gyrus L	0.072,0.086,0.068	0.055,0.082,0.055	0.066,0.076,0.06
Lingual gyrus R	0.112,0.098,0.093	0.044,0.079,0.048	0.11,0.089,0.087
Inferior occipital lobe L	0.058,0.101,0.069	0.056,0.069,0.055	0.052,0.087,0.061
Inferior occipital lobe R	0.031,0.048,0.034	0.117,-0.021,0.06	0.015,0.048,0.025
Middle occipital lobe L	0.02,0.086,0.044	0.062,0.072,0.061	0.011,0.075,0.035
Middle occipital lobe R	0.055,0.084,0.063	0.094,0.048,0.07	0.042,0.072,0.053
Superior occipital lobe L	0.066,0.117,0.083	0.05,0.047,0.046	0.058,0.11,0.077
Superior occipital lobe R	0.06,0.127,0.083	0.085,0.013,0.056	0.045,0.121,0.076
Pallidum L	0.034,0.104,0.054	0.09,-0.001,0.051	0.022,0.11,0.047
Pallidum R	0.033,0.111,0.057	0.067,-0.044,0.025	0.025,0.124,0.054
Paracentral lobule L	0.062,0.038,0.052	0.041,0.069,0.049	0.064,0.027,0.046
Paracentral lobule R	0.073,0.101,0.082	0.054,-0.001,0.032	0.071,0.102,0.078
Parahippocampal gyrus L	0.073,0.122,0.077	0.056,0.049,0.044	0.066,0.115,0.071
Parahippocampal gyrus R	0.104,0.115,0.091	0.032,0.093,0.045	0.103,0.101,0.086

Inferior parietal lobule L	0.069,0.021,0.052	0.024,0.082,0.046	0.069,0.009,0.047
Inferior parietal lobule R	0.032,0.041,0.037	0.095,0.104,0.095	0.024,0.026,0.024
Superior parietal lobule R	0.104,0.054,0.086	0.044,0.082,0.057	0.102,0.043,0.08
Superior parietal lobule L	0.108,0.069,0.092	0.055,0.058,0.055	0.106,0.059,0.086
Postcentral gyrus L	0.063,0.106,0.08	0.103,0.101,0.099	0.051,0.092,0.066
Postcentral gyrus R	0.085,0.06,0.076	0.034,0.068,0.046	0.083,0.051,0.07
Precentral gyrus L	0.096,0.021,0.066	0.045,0.046,0.044	0.097,0.013,0.061
Precentral gyrus R	0.07,0.108,0.08	0.088,0.073,0.077	0.062,0.097,0.069
Precuneus L	0.127,0.088,0.106	0.045,0.064,0.048	0.125,0.079,0.101
Precuneus R	0.112,0.138,0.115	0.049,0.098,0.062	0.109,0.125,0.107
Putamen L	0.02,0.097,0.048	0.074,-0.051,0.025	0.01,0.109,0.045
Putamen R	0.014,0.117,0.051	0.08,-0.018,0.039	0.004,0.124,0.045
Rectus gyrus L	0.111,0.109,0.104	0.05,0.103,0.063	0.109,0.095,0.095
Rectus gyrus R	0.136,0.09,0.111	0.049,0.106,0.063	0.134,0.075,0.103
Rolandic operculum L	0.086,0.065,0.077	0.109,0.089,0.096	0.072,0.052,0.063
Rolandic operculum R	0.101,0.131,0.11	0.094,0.092,0.088	0.09,0.12,0.097

Supplemental motor area L	0.032,0.048,0.038	0.035,0.076,0.047	0.034,0.044,0.032
Supplemental motor area R	0.053,0.055,0.053	0.062,0.059,0.057	0.051,0.049,0.045
Supramarginal gyrus L	0.072,0.029,0.056	0.05,0.042,0.044	0.071,0.021,0.051
Supramarginal gyrus R	0.025,0.106,0.053	0.08,0.075,0.072	0.019,0.096,0.044
Inferior temporal gyrus L	0.052,0.06,0.047	0.071,0.064,0.057	0.045,0.049,0.039
Inferior temporal gyrus R	0.04,0.08,0.046	0.04,0.047,0.034	0.038,0.076,0.042
Middle temporal gyrus L	0.065,0.081,0.066	0.058,0.076,0.057	0.06,0.07,0.058
Middle temporal gyrus R	0.06,0.104,0.067	0.059,0.111,0.066	0.054,0.09,0.058
Temporal pole middle part L	0.037,-0.007,0.021	0.046,0.069,0.049	0.031,-0.017,0.013
Temporal pole middle part R	0.042,0.037,0.037	0.027,0.027,0.024	0.039,0.033,0.034
Temporal pole superior part L	0.087,0.059,0.077	0.106,0.042,0.078	0.071,0.055,0.066
Temporal pole superior part R	0.088,0.121,0.096	0.066,0.049,0.055	0.078,0.117,0.089
Superior temporal gyrus L	0.116,0.08,0.099	0.095,0.058,0.076	0.104,0.072,0.088
Superior temporal gyrus R	0.091,0.1,0.087	0.084,0.06,0.068	0.082,0.091,0.078
Thalamus L	0.122,0.052,0.092	0.031,-0.001,0.019	0.119,0.06,0.091
Thalamus R	0.142,0.056,0.105	0.055,0.005,0.035	0.136,0.062,0.102

Cerebellum L	0.028,0.061,0.037	0.043,0.037,0.034	0.026,0.054,0.031
Cerebellum R	0.047,0.067,0.049	0.073,0.036,0.051	0.041,0.062,0.042

References

1. Takeuchi, H. et al. The association between resting functional connectivity and creativity. *Cereb. Cortex* **22**, 2921-2929 (2012).
2. Raven, J. Manual for Raven's progressive matrices and vocabulary scales. (Oxford Psychologists Press Oxford, 1993).
3. Takeuchi, H. et al. White matter structures associated with creativity: Evidence from diffusion tensor imaging. *Neuroimage* **51**, 11-18 (2010).
4. Takeuchi, H. et al. Regional gray matter volume of dopaminergic system associate with creativity: Evidence from voxel-based morphometry *Neuroimage* **51**, 578-585 (2010).
5. Takeuchi, H. et al. Failing to deactivate: the association between brain activity during a working memory task and creativity. *Neuroimage* **55**, 681-687 (2011).
6. Hakoda, Y. & Sasaki, M. Group version of the Stroop and reverse-Stroop Test : The effects of reaction mode, order and practice. *Kyoikushinrigakukenkyu (Educational Psychology Research)* **38**, 389-394 (1990).
7. Takeuchi, H. et al. Regional gray and white matter volume associated with Stroop interference: Evidence from voxel-based morphometry. *Neuroimage* **59**, 2899-2907 (2012).
8. Tanaka, K., Okamoto, K. & Tanaka, H. Manual of New Tanaka B type intelligence test. (Kaneko Syobo, Tokyo, 2003).
9. Takeuchi, H. et al. Effects of training of processing speed on neural systems. *J. Neurosci.* **31**, 12139-12148 (2011).
10. Maldjian, J.A., Laurienti, P.J. & Burdette, J.H. Precentral gyrus discrepancy in electronic versions of the Talairach atlas. *Neuroimage* **21**, 450-455 (2004).

11. Maldjian, J.A., Laurienti, P.J., Kraft, R.A. & Burdette, J.H. An automated method for neuroanatomic and cytoarchitectonic atlas-based interrogation of fMRI data sets. *Neuroimage* **19**, 1233-1239 (2003).

12. Tzourio-Mazoyer, N. et al. Automated anatomical labeling of activations in SPM using a macroscopic anatomical parcellation of the MNI MRI single-subject brain. *Neuroimage* **15**, 273-289 (2002).
